# Supplementary material for: Cross-sectional study of nutritional intake among patients undergoing tuberculosis treatment along the Myanmar–Thailand border
Source: BMJ Open. 2022 Jan 6;12(1):e052981. doi: 10.1136/bmjopen-2021-052981 (PMC8744095; doi:10.1136/bmjopen-2021-052981)
Supplement: Author's manuscript [file bmjopen-2021-052981.draft_revisions.pdf]

BMJ Open is committed to open peer review. As part of this commitment we make the peer review history of every article we publish publicly available.

When an article is published we post the peer reviewers' comments and the authors' responses online. We also post the versions of the paper that were used during peer review. These are the versions that the peer review comments apply to.

The versions of the paper that follow are the versions that were submitted during the peer review process. They are not the versions of record or the final published versions. They should not be cited or distributed as the published version of this manuscript.

BMJ Open is an open access journal and the full, final, typeset and author-corrected version of record of the manuscript is available on our site with no access controls, subscription charges or pay-per-view fees (http://bmjopen.bmj.com).

If you have any questions on BMJ Open's open peer review process please email info.bmjopen@bmj.com

A cross-sectional study of nutritional intake among patients undergoing tuberculosis treatment along the Myanmar-Thailand border

Journal:	*BMJ Open*
Manuscript ID	bmjopen-2021-052981
Article Type:	Original research
Date Submitted by the Author:	02-May-2021

Complete List of Authors:	Damji, Karim; California State University Northridge<br>Hashmi, Ahmar; Chiang Mai University, Department of Family Medicine<br>Kyi, Lin Lin; Mahidol Oxford Tropical Medicine Research Unit, Shoklo Malaria Research Unit<br>Vincenti-Delmas, Michele; Mahidol Oxford Tropical Medicine Research Unit, Shoklo Malaria Research Unit<br>Htun, Win Pa Pa; Mahidol Oxford Tropical Medicine Research Unit, Shoklo Malaria Research Unit<br>Ko Ko Aung, Htet; Mahidol Oxford Tropical Medicine Research Unit, Shoklo Malaria Research Unit<br>Brummaier, Tobias; Mahidol Oxford Tropical Medicine Research Unit; Swiss Tropical and Public Health Institute<br>Angkurawaranon, Chaisiri; Faculty of Medicine, Chiang Mai University, Department of Family Medicine<br>Carrara, Verena; Mahidol Oxford Tropical Medicine Research Unit, Shoklo Malaria Research Unit<br>Nosten, Francois; Centre for Tropical Medicine and Global Health, Nuffield Department of Medicine, University of Oxford; Shoklo Malaria Research Unit, Mahidol-Oxford Tropical Medicine Research Unit, Faculty of Tropical Medicine, Mahidol University

Keywords:	Tuberculosis < INFECTIOUS DISEASES, NUTRITION & DIETETICS, EPIDEMIOLOGY, Tropical medicine < INFECTIOUS DISEASES

I, the Submitting Author has the right to grant and does grant on behalf of all authors of the Work (as defined in the below author licence), an exclusive licence and/or a non-exclusive licence for contributions from authors who are: i) UK Crown employees; ii) where BMJ has agreed a CC-BY licence shall apply, and/or iii) in accordance with the terms applicable for US Federal Government officers or employees acting as part of their official duties; on a worldwide, perpetual, irrevocable, royalty-free basis to BMJ Publishing Group Ltd ("BMJ") its licensees and where the relevant Journal is co-owned by BMJ to the co-owners of the Journal, to publish the Work in this journal and any other BMJ products and to exploit all rights, as set out in our licence.

The Submitting Author accepts and understands that any supply made under these terms is made by BMJ to the Submitting Author unless you are acting as an employee on behalf of your employer or a postgraduate student of an affiliated institution which is paying any applicable article publishing charge ("APC") for Open Access articles. Where the Submitting Author wishes to make the Work available on an Open Access basis (and intends to pay the relevant APC), the terms of reuse of such Open Access shall be governed by a Creative Commons licence – details of these licences and which Creative Commons licence will apply to this Work are set out in our licence referred to above.

Other than as permitted in any relevant BMJ Author's Self Archiving Policies, I confirm this Work has not been accepted for publication elsewhere, is not being considered for publication elsewhere and does not duplicate material already published. I confirm all authors consent to publication of this Work and authorise the granting of this licence.

For peer review only

A cross-sectional study of nutritional intake among patients undergoing tuberculosis treatment along the Myanmar-Thailand border

Karim Damji[1], Ahmar H Hashmi[2]*, Lin Lin Kyi[3], Michele Vincenti-Delmas[3], Win Pa Pa Htun[3], Htet Ko Ko Aung[3], Tobias Brummaier[3,4,5,6], Chaisiri Angkurawaranon[2], Verena I Carrara[3,4], Francois Nosten[3,4]

[1]Family and Consumer Sciences, California State University, Northridge, USA

[2]Faculty of Medicine, Chiang Mai University, Chiang Mai, Thailand

[3]Shoklo Malaria Research Unit, Mahidol-Oxford Tropical Medicine Research Unit, Mae Sot, Thailand

[4] Centre for Tropical Medicine and Global Health, University of Oxford, Oxford, UK

[5]Swiss Tropical and Public Health Institute, Basel, Switzerland

[6]University of Basel, Basel, Switzerland

*Corresponding author: ahmar.hashmi715@gmail.com; Department of Family Medicine, Faculty of Medicine, Chiang Mai University, 110 Intharavoros Road, Chiang Mai 50220 Thailand

Karim Damji: karim.damji.945@my.csun.edu

Ahmar H Hashmi: ahmar.hashmi715@gmail.com

Lin Lin Kyi: nanlinlinkyi33@gmail.com

Michele Vincenti-Delmas: michele.vincenti@gmail.com

Win Pa Pa: winpapa@shoklo-unit.com

Htet Ko Ko Aung: htetkoko@shoklo-unit.com

Tobias Brummaier: tobias@shoklo-unit.com

Chaisiri Angkurawaranon: chaisiri.a@cmu.ac.th

Verena I Carrara: verena@shoklo-unit.com

Francois Nosten: francois@tropmedres.ac

For peer review only

Abstract

Objective: This study summarizes nutritional intake among TB patients along the Myanmar-Thailand border according to the local diet.

Setting: TB sanatorium along the Myanmar-Thailand border.

Participants: Patients receiving anti-tuberculous treatment were enrolled for a 24 hour food recall in cross-section. Participants were selected to reflect proportion of age, sex, and HIV co-infection based on historical patient records. Out of a total of 28 participants, 20 (71.4%) were male and 5 (17.9%) were co-infected with HIV.

Primary and secondary outcome measures: Actual vs recommended nutrition intake; weight gain.

Results: There were no significant differences in macro- or micronutrient intake when stratified by sex or for patients supplementing meal rations. Mean duration of treatment at time of survey was 20.7 weeks (95% CI: 16.54, 24.82). A significantly higher proportion of women (8/8, 100%) met caloric requirements compared to men (9/20, 45.0%, p=0.010), but few participants met other macro- or micronutrient requirements, with no significant differences by sex or for patients supplementing their rations. From diagnosis to the time of the survey, although participants had significant weight gain averaging 6.48 kg (95% CI: 3.87, 9.10) and 2.47 kg/m^2 (95% CI: 1.45, 3.49) in BMI (p=0.0001 for both), 50% (14/28) still had mild or more severe forms of malnutrition.

Conclusions: This cross-sectional survey of TB patients residing in a sanatorium demonstrates the difficulty in sufficiently meeting nutritional demands, even when providing nutritional support.

Keywords: tuberculosis; malnutrition; nutrition assessment; nutritional support; transients and migrants;

Word count: 2470 words

For peer review only

Article Summary

Strengths and limitations of tise study

- Strong methodology tailored to the local diet to accurately assess nutritional intake;
- Adds to the literature with findings for a marginal and vulnerable population for which accurate data is lacking;
- Limited in sample size and other more costly measures that may more precisely estimate nutritional intake.

For peer review only

Introduction

The interaction of malnutrition and tuberculosis (TB) has been long established [1]. Not only does malnutrition predispose to TB infection, poor nutritional status and weight loss—particularly loss of lean body mass—are also associated with TB [2-4]. Malnutrition at the time of TB diagnosis increases mortality risk [2 5 6]. Inadequate nutritional intake is most likely the cause of poor nutritional status among patients with TB [1]. However, there is insufficient evidence to determine whether nutritional supplementation improves outcomes in patients with TB [3 7 8]. Even in situations where anti-tuberculous therapy and nutritional support is offered, many patients do not return to adequate nutritional status by the end of their treatment period [1 9]. But in light of the strong link between malnutrition and TB, the WHO insists on nutritional assessment, counseling, and addressing nutritional deficits for patients with TB [3 5 8 10 11].

However, it is difficult to assess nutritional intake of patients with TB to detect underlying nutritional deficits. Nutritional assessments commonly use 24-hour food recalls or food frequency questionnaires. In low- and middle-income countries—where the bulk of global TB cases occur—the lack of nutrient composition data for local diets leads to inaccurate estimates of nutrient intake [11]. These intake assessments relying on patient recall may be limited when anti-tuberculous treatment is provided as outpatient or community directly observed therapy (DOTS) programs [2 10].

Along the Myanmar-Thailand border, TB clinics provide services for migrants with TB via a unique, sanatorium model [12-14]. Nutritional support, in the way of rations, are included in the cadre of care and treatment services provided to patients who reside at the clinics for the duration of their 3- to 6-month treatment. Recent nutrition research over the past few years has allowed a better characterization of the local diets along the border sufficient to determine nutritional content [15 16]. This retrospective, cross-sectional study seeks to quantify

For peer review only

macro- and micro-nutritional intake of a sample of patients with TB along the border and to evaluate if meal rations adequately address patients' nutritional demands.

Methods

Setting: Tuberculosis Program along the border

Established in 2010, the Tuberculosis Program at the Borderlands Health Foundation/Shoklo Malaria Research Unit (SMRU) has provided a large breadth of services for TB patients through its sanatorium, including diagnostic and treatment procedures and also housing and accommodation, dietary, and psychosocial support services. Patients are provided daily meals for the duration of their treatment at the sanatorium. By the end of January 2019, these clinics had provided treatment services to a total of 1925 patients, 1465 (76%) of whom had successfully completed the entirety of their treatment. At the time of this study, 118 patients were actively receiving treatment. Patients are agricultural migrants originally from either side of the Myanmar-Thailand border, predominantly of Burman or Karen ethnicity.

For peer review only

Study participants and design

Stratified sampling was performed to select patients from participating TB clinics. TB program patient records were reviewed to estimate the proportion of men and women, age, and those co-infected with HIV among the patient population. These proportions were then applied to the existing roster of patients currently under care and screened to determine eligibility. Participants included had TB confirmed by Gene Xpert, with pulmonary or extra pulmonary infections, multi-drug resistant TB, or HIV co-infection. Routine treatment was provided to all patients according to WHO guidelines. Screened participants were excluded if they were in treatment for less than 8 weeks, those with recent acute onset of illness, those unable to provide accurate 24-hour food recalls, or those unable to provide consent.

Survey tools and validation

24-hour food recalls were used to determine participant food intake, which was adapted from a form validated for use for an observational cohort, "Molecular signature of Karen and

For peer review only - http://bmjopen.bmj.com/site/about/guidelines.xhtml

Burmese pregnant women on the Thailand-Myanmar border," conducted at SMRU (ClinicalTrials.gov Identifier: NCT02797327) [15]. However, as portion size estimations—using visual and other size estimation aids or household utensil measures—are limited[17-19], caloric intake is often underestimated, such as for cereal grains among other food items [19]. As a response, dietitians advocate the use of alternative methods including hand measurements to estimate portion sizes in 24-hour food recalls, which has been validated for accuracy and appropriateness of this method for use in low resource settings [15]. In previous 24-hour food recalls in migrant clinics along the Myanmar-Thailand border, patients often underestimated their intake of rice—similar to studies in Africa [19]. Therefore, 24-hour food recalls have employed all methods at our disposal: standardized measuring instruments, hand measurements, as well as visual aids [17]. Using multiple methods of portion size estimation may enhance the accuracy of recall data.

Calculating Nutritional Intake

Following survey completion, food recall data was uploaded into an online nutrition data software (Cronometer, https://cronometer.com/). Nutrition data was calculated for the total intake, intake from rations provided and intake excluding rice.

Rations provided by the sanatorium included two full meals a day as well as refreshments (processed foods and liquids). Some participants were able to supplement the rations provided with food from outside sources which were also captured on 24-hour food recalls.

Recipes for all meals provided at the sanatorium as part of the rations were gathered through interviewing the cooks at the sanatorium and corroborating common recipes with several local staff at SMRU. This allowed compiling basic recipes for curries, soups, salads, stir fries, and pastes. Any dish or meal recalled as part of the rations allowed for the main ingredients to be determined and used in conjunction with basic recipes. These final recipes were then added to Cronometer.

For peer review only - http://bmjopen.bmj.com/site/about/guidelines.xhtml

As several ingredients in this community's diet were not available on Cronometer's database, nutrient data was clarified using the Mahidol University's Thai Food Composition Database or McGill University's Center for Indigenous Peoples' Nutrition and Environment Karen food composition database [20 21]. Nutritional information in these databases is often expressed in terms of nutrients per 100-gram sample, hence densities of ingredients was required. This was determined either through direct measurement, matching densities to foods with similar descriptions, or averaging the densities of similar ingredients.

Anthropometric measurements and determining nutritional requirements

Anthropometric measurements were performed for each participant immediately prior to conducting the 24-hour food recall and followed TB Program standard of procedures for weight and height. Routinely calibrated mechanical scales were used to measure weight and height accurate to 500 grams and 5 mm, respectively. BMI was calculated and subdivided into: severe malnutrition (< 16.0 kg/m^2), moderate malnutrition (16.0 – 16.9 kg/m^2), mild malnutrition (17.0 – 18.4 kg/m^2), normal (18.5 – 24.9 kg/m^2), and overweight (25.0 – 29.9 kg/m^2).

Nutritional requirements for comparison against participants' actual intake were calculated for each individual patient. For TB patients without comorbidities, we used a protein range of 1.2-1.5 g/kg Ideal Body Weight (IBW). The energy range was 35-40 kcal/kg body weight. IBW was calculated using the Devine Equation (50kg +2.3kg X (Ht – 60 in) for men, and 45.5kg +2.3kg X (Ht - 60 in) for women), which was adapted for the metric system [22]. For patients with an HIV coinfection, the 35-40 kcal/kg range was increased by 20-30%. The estimated energy needs of the one participant with an above-the-knee amputation were reduced by 16%. National Academy of Medicine recommendations were used for carbohydrates and fats [23] and the Thai daily recommended intake (DRI) for micronutrients (vitamins A, B-complex, C, calcium, folate, iron, sodium, zinc) [24].

For peer review only - http://bmjopen.bmj.com/site/about/guidelines.xhtml

Statistical Analysis

Analysis was conducted using Stata version 14 (StataCorp LP, College Station, TX, USA). Data were assessed for normality, and descriptive data were summarized using mean and standard deviation or median and range as appropriate. The primary outcome of this study was to compare participants' dietary intake with their nutritional requirements. Participant intake is summarized for all three macronutrients (protein, carbohydrates, and fats) and six selected micronutrients. To allow for comparisons, these data were stratified according to sex and whether or not participants supplemented their rations with additional food. Given the normality of the data, statistical comparisons were made using Student's t-test, Mann-Whitney U test, Wilcoxon signed rank test, or Chi-squared test according to continuous or categorical variables. Sex, age, BMI at diagnosis, and duration of treatment was assessed for potential association with outcomes of interest.

Patient and public involvement in research

This study was initially begun as an audit of nutrition services provided at the TB clinic to improve patient diet. Although patients were satisfied overall, we attempted to understand if nutrition provided actual met their required needs. Study findings will be disseminated to both patients and staff at the clinics. In addition, the research team engaged the local population through a local community advisory board, the Tak Community Advisory Board, Thailand. Comprised of community leaders, they advised on study design and implementation and approved the study (TCAB 201903).

Results

Table 1 highlights basic participants' information for a total of 28 participants. More men were included in the analysis (20/28, 71.4% vs 8/28, 28.6%) and were significantly older than women (52.4 vs 37.6 years, p=0.021). A total of 17.9% (5/28) of participants were coinfected with HIV. Just over half the participants (57.1%, 16/28) ate only the rations provided by the clinic. At diagnosis, men were significantly taller (160.7 vs 150.8 cm, p=0.002) and heavier

For peer review only - http://bmjopen.bmj.com/site/about/guidelines.xhtml

(43.2 vs 36.9 kg, p=0.011) than women, but there were no significant differences for BMI. Mean duration of treatment at the time of survey was 20.7 weeks (95% CI: 16.5-24.8). Pairwise comparisons show 22/28 (78.6%) participants had increased weight and BMI from diagnosis to the time of the survey; with an average gain of 6.48 kg (95% CI: 3.87, 9.10) and 2.47 kg/m^2 (95% CI: 1.45, 3.49) per participant (p=0.0001 for both). By the time of the survey, weight of men and women were no longer significantly different (51.0 vs 44.0 kg, p=0.115). This improvement in weight and BMI is reflected in the shift of malnutrition category from diagnosis to the time of survey: at the time of diagnosis 89% (25/28) of participants had mild or more severe forms of malnutrition compared to 50% (14/28) of participants at the time of the survey (p=0.22).

Table 2 outlines the average nutritional intake stratified by sex and whether participants ate rations only. There were no significant differences in total nutritional intake for macro- or micronutrient when comparing men to women, or patients eating only the rations provided compared to those supplementing the rations received.

Table 3 highlights the proportion of participants meeting their recommended daily allowances. Participants' diets were low in protein, fats, and nearly all vitamins, but were high in salt content. A significantly higher proportion women met their overall caloric and vitamin C requirements (p=0.010, p=0.044), while a higher proportion of men met their iron intake requirements (p=0.011). Meeting the remaining dietary requirements did not differ significantly between the sexes or having a ration-only diet.

Discussion

This study presents findings from a cross-sectional survey of TB patients undergoing treatment while residing at a sanatorium along the Myanmar-Thailand border. The most notable finding in this study is the difficulty patients had reaching their nutrition requirements. Even TB patients supplementing the rations provided by the clinic were not

For peer review only - http://bmjopen.bmj.com/site/about/guidelines.xhtml

significantly more likely to meet their requirements than those eating only the provided rations. Total calories and protein requirements were often not met, and although carbohydrates approached the needed requirements, that most of the total carbohydrates were acquired from rice reflects a lack of dietary diversity. This lack of dietary diversity likely relates to participants' being unable to meet most micronutrient requirements. Similar to other study findings, although there was some weight gain and improvement in BMI, and malnutrition status, this was not to the level desired [1 8 9]. Overall intake of almost all important micronutrients was limited, with sodium intake often being too high—even for patients eating only the rations provided. This is most likely due to the limited diversity represented in the patients' diets.

These inadequacies in nutrient intake among TB patients echoes findings in other studies [1 2 7 9 25]. This study adds to the literature by including robust data on the nutritional content of local diets; provides strong nutritional intake data given the sanatorium setting; and provides baseline data for a vulnerable, migrant population that likely has worse nutritional status upon diagnosis than may be seen in other populations. Unlike another study from Georgia where nutrient intake was estimated in accordance to the local diet, TB patients in Georgia were able to manage high caloric intake for follow-up in an outpatient setting [10].

In this setting in Asia, it may be useful to highlight some recommendations for increasing nutritional intake: a regular source of protein (animal-based if possible); small yet regular meals; and locally sourced fresh fruits and vegetables or other snacks low in sodium and saturated fats that provide additional micronutrient supplementation. These recommendations should be considered alongside regular dietary counseling and support—if human resources can be allocated—as recommended by the WHO [8]. Not only are patients recommended to have an assessment at intake, but support may also be important at the beginning stages of drug treatment, when side effects such as nausea, vomiting, and loss of appetite can interfere

For peer review only - http://bmjopen.bmj.com/site/about/guidelines.xhtml

with early nutritional gains [25]. A culturally-tailored counseling program, representative of the local diet, can help inform TB patients about the importance of a diverse diet, especially when undergoing treatment and as local community awareness about good nutrition may be limited. Given the sanatorium setting, including family members in these discussions would be helpful. Such counseling can also help inform TB patients (and their caregivers) of good nutritional foods should they be able to purchase foods to supplement their rations.

This study is not without limitations. It is cross-sectional in design—although this is the design reported by most studies of this nature. With further funding, future studies will assess nutritional intake in addition to blood and serum measures of nutritional status among a larger sample of patients. The comparisons drawn in this sample are limited as well, given the broad inclusion criteria (e.g., including HIV co-infected or multi-drug resistant TB cases) that were chosen to include a more representative sample of patients from this border region to inform future nutritional interventions. Future studies may also benefit from using additional anthropometric measures, especially those more sensitive in detecting undernutrition [6 9 26 27] and that account for disease severity [2 5]. Some limitation is inherent to the methods used such as measurement intake accuracy [17 18] and recall bias, and aligning food descriptions from the local diet with food composition databases, but the tools used have been developed across different nutritional studies in this area and have allowed for validating methods across different segments of the local migrant populations.

Conclusions

This cross-sectional survey of TB patients residing in a sanatorium along the Myanmar-Thailand border demonstrates the difficulty in sufficiently meeting nutritional demands, even when providing nutritional support.

Abbreviations

BMI	Body-mass index
DRI	Daily recommended intake

For peer review only

SMRU Shoklo Malaria Research Unit
TB Tuberculosis
WHO World Health Organization

260 **Acknowledgements**

261 We are grateful for the patients who volunteered to be a part of this study. We would like to
262 acknowledge the staff working in the TB department at SMRU, for their help and support
263 with logistics, materials, and other needs. We would like to thank the local SMRU staff that
264 helped with recipes and understanding local diets, including the cooks at the TB sanatorium.
265 We would like to thank Wolfgang Stuetz for feedback on drafts of this manuscript.

266 **Ethics approval**

267 This study received ethical approval from the Research Ethics Committee of the Faculty of
268 Medicine, Chiang Mai University (FAM-2562-06197) in accordance with the Declaration of
269 Helsinki. Informed verbal assent was given by all participants prior to survey, with written
270 informed consent waived by the Research Ethics Committee of Chiang Mai University.

271 **Consent for publication**

272 Not applicable.

273 **Data availability statement**

274 The datasets generated and/or analyzed during the current study are not publicly available
275 due to privacy/safety for migrant participants, but are available from the corresponding
276 author on reasonable request.

277 **Competing interests**

278 The authors declare that they have no competing interests.

279 **Funding**

280 This research was funded in whole, or in part, by the Wellcome Trust Thailand/Laos Major
281 Overseas Program Renewal [grant number 106698]. For the purpose of open access, the
282 author has applied a CC BY public copyright licence to any Author Accepted Manuscript

For peer review only

For peer review only - http://bmjopen.bmj.com/site/about/guidelines.xhtml

version arising from this submission. The funding body had no role in the design of the study and collection, analysis, and interpretation of data and in writing the manuscript.

Author contributions

KD, AHH, MVD, CA, VIC, FN: study conception, design; KD, AHH, LLK, WPPH, HKKA, TB: data collection and study implementation; KD, AHH, VIC: data analysis/interpretation; All authors critically revised the manuscript and approved the final version submitted and agree to be accountable for this work.

References

1. Macallan DC. Malnutrition in tuberculosis. *Diagnostic microbiology and infectious disease* 1999;34(2):153-7.
2. Mupere E, Parraga IM, Tisch DJ, et al. Low nutrient intake among adult women and patients with severe tuberculosis disease in Uganda: a cross-sectional study. *BMC Public Health* 2012;12:1050.
3. Sinclair D, Abba K, Grobler L, et al. Nutritional supplements for people being treated for active tuberculosis. *Cochrane Database of Systematic Reviews* 2011;11:CD006086.
4. White LV, Edwards T, Lee N, et al. Patterns and predictors of co-morbidities in Tuberculosis: A cross-sectional study in the Philippines. *Scientific Reports* 2020;10:4100.
5. Miyata S, Tanaka M, Ihaku D. The prognostic significance of nutritional status using malnutrition universal screening tool in patients with pulmonary tuberculosis. *Nutr J* 2013;12:42.
6. Zachariah R, Spielmann MP, Harries AD, et al. Moderate to severe malnutrition in patients with tuberculosis is a risk factor associated with early death. *Transactions of the Royal Society of Tropical Medicine and Hygiene* 2002;96(3):291-4.
7. Rudolph M, Kroll F, Beery M, et al. A pilot study assessing the impact of a fortified supplementary food on the health and well-being of creche children and adult TB patients in South Africa. *PLoS ONE [Electronic Resource]* 2013;8(1):e55544.
8. World Health Organization. Nutritional care and support for patients with tuberculosis. Geneva: WHO, 2013.
9. Bacelo AC, Ramalho A, Brasil PE, et al. Nutritional supplementation is a necessary complement to dietary counseling among tuberculosis and tuberculosis-HIV patients. *PloS one* 2015;10(8):e0134785.
10. Frediani JK, Sanikidze E, Kipiani M, et al. Macronutrient intake and body composition changes during anti-tuberculosis therapy in adults. *Clin Nutr* 2016;35(1):205-12.
11. Frediani JK, Tukvadze N, Sanikidze E, et al. A culture-specific nutrient intake assessment instrument in patients with pulmonary tuberculosis. *Clin Nutr* 2013;32(6):1023-8.
12. Thi SS, Parker DM, Swe LL, et al. Migration histories of multidrug-resistant tuberculosis patients from the Thailand-Myanmar border, 2012-2014. *Int J Tuberc Lung Dis* 2017;21(7):753-58.
13. Tschirhart N, Nosten F, Foster AM. Migrant tuberculosis patient needs and health system response along the Thailand-Myanmar border. *Health Policy Planning* 2017;32:1212-19.

For peer review only

14. Tschirhart N, Sein T, Nosten F, et al. Migrant and refugee patient perspectives on travel and tuberculosis along the Thailand-Myanmar border: a qualitative study. *PLOS ONE* 2016;11(8):e0160222.
15. Brummaier T, Kabeer BSA, Lindow S, et al. A prospective cohort for the investigation of alteration in temporal transcriptional and microbiome trajectories preceding preterm birth: a study protocol. *BMJ Open* 2018;9:e023417.
16. Brummaier T, Kabeer BSA, Wilaisrisak P, et al. Cohort profile: molecular signature in pregnancy (MSP): longitudinal high-frequency sampling to characterise cross-omic trajectories in pregnancy in a resource-constrained setting. *BMJ Open* 2020;10:e041631.
17. Amoutzopoulos B, Paige P, Roberts C, et al. Portion size estimation in dietary assessment: a systematic review of existing tools, their strengths and limitations. *Nutrition Reviews* 2020;78(11)
18. Gibson AA, Hsu MS, Rangan AM, et al. Accuracy of hands *v.* household measures as portion size estimation aids. *J Nutritional Sci* 2016;5(e29)
19. Ogunbode AM, Owolabi MO, Ogunbode OO, et al. What's in your hands? A systematic review of dietary assessment methods and estimation of food sizes in a primary care clinic. *J Med Tropics* 2018;20(2):93.
20. Mahidol University, Institute of Nutrition. Thai Food Composition Database 2015 [Available from: http://www.inmu.mahidol.ac.th/thaifcd.
21. McGill University, Environment CfIPNa. CINE Global Health Case Study - Karen 2019 [Available from: https://www.mcgill.ca/cine/resources/data/karen.
22. Peterson CM, Thomas DM, Blackburn GL, et al. Universal equation for estimating ideal body weight and body weight at any BMI. *Am J Clin Nutr* 2016;103(5):1197-203.
23. National Academy of Medicine. Dietary reference intakes for energy, carbohydrate, fiber, fat, fatty acids, cholesterol, protein, and amino acids. Washington, D.C.: National Academy of Medicine, 2005.
24. Bureau of Nutrition, Department of Health, Thailand Ministry of Public Health. Dietary reference intake for Thais 2020. Bangkok: Ministry of Public Health, 2020.
25. Bacelo AC, do Brasil PE, Cople-Rodrigues CD, et al. Dietary counseling adherence during tuberculosis treatment: A longitudinal study. *Clin Nutr ESPEN* 2017;17:44-53.
26. Lee N, White LV, Marin FP, et al. Mid-upper arm circumference predicts death in adult patients admitted to a TB ward in the Philippines: a prospective cohort study. *PLOS ONE* 2019;14(6):e0218193.
27. White LV, Lee N, Marin FP, et al. Performance of alternative measures to body mass index in the assessment of moderate and severe under-nutrition among acutely unwell patients hospitalized in a TB ward in the Philippines: a cross-sectional study. *PLOS ONE* 2019;14(5):e0215968.

For peer review only - http://bmjopen.bmj.com/site/about/guidelines.xhtml

Table 1. Basic demographic, treatment, and anthropometric variables.

Participants (N=28)		
Male[1]	20 (71.4%)	
Female	8 (28.6%)	
Age (mean, 95% CI, years)	48.18 (42.11, 54.24)	p=0.021
Male	52.40 (45.86, 58.94)	
Female	37.62 (24.79, 50.46)	
Ration only (n, %)		
Yes	16 (57.1)	
No	12 (42.9)	
HIV coinfection (n, %)		
Yes	5 (17.9)	
No	23 (82.1)	
Duration of treatment (mean, 95% CI, weeks)	20.68 (16.54, 24.82)	p=0.628
Male	20.08 (15.41, 24.75)	
Female	22.18 (11.41, 32.94)	
Height (mean, 95% CI, cm)	157.8 (154.6, 161.1)	p=0.003
Male	160.7 (157.4, 163.9)	
Female	150.8 (144.4, 157.2)	
Weight at diagnosis (mean, 95% CI, kg)	41.4 (39.1, 43.8)	p=0.011
Male	43.2 (40.7, 45.8)	
Female	36.9 (31.9, 41.8)	
Weight at survey (mean, 95% CI, kg)	49.0 (44.9, 53.0)	p=0.115
Male	51.0 (46.3, 55.6)	
Female	44.0 (35.1, 52.9)	
BMI at diagnosis (mean, 95% CI, kg/m^2)	16.1 (15.7, 17.5)	p=0.630
Male	16.7 (15.7, 17.8)	
Female	16.3 (13.8, 18.7)	
BMI at survey (mean, 95% CI, kg/m^2)	19.5 (18.3, 20.7)	p=0.682
Male	19.7 (18.3, 21.1)	
Female	19.1 (16.3, 21.9)	

[1]Includes one participant with an amputation of one leg.

Table 2. Nutrition intake reported as median (IQR).

	Totals (N=28)	Male (n=20)	Female (n=8)	Ration only (n=16)	Ration+ (n=12)
Calories (kcal)	2153.5 (1764.0, 3118.0)	2065.0 (1487.5, 3118.0)	2325.0 (1986.5, 3158.5)	2035.0 (1454.5, 2721.5)	2153.5 (1968.0, 3638.0)
Calories from rice (kcal)	967.0 (789.0, 1396.5)	967.0 (842.0, 1450.5)	967.0 (698.5, 1396.5)	967.0 (644.5, 1397.0)	967.5 (789.0, 1396.5)
Protein (kcal)	277.6 (192.6, 371.6)	268.6 (178.4, 371.6)	305.8 (225.8, 421.4)	268.6 (163.6, 360.8)	302.4 (225.8, 422.0)
Carbohydrates (kcal)	1240.4 (1061.6, 1718.0)	1248.0 (1025.6, 1891.0)	1231.8 (1098.0, 1522.0)	1160.2 (1008.6, 1718.0)	1313.8 (1173.8, 1791.8)
Fats (kcal)	540.9 (385.7, 797.4)	455.9 (383.9, 736.7)	756.9 (526.5, 982.4)	464.9 (356.4, 711.0)	736.7 (403.2, 947.7)

For peer review only

A (mcg RAE)	293.8 (150.5, 459.4)	226.8 (150.5, 459.4)	360.7 (150.6, 494.2)	226.8 (1510.5, 425.6)	398.8 (141.9, 502.6)
B1 (mg)	1.0 (0.7, 1.5)	1.0 (0.8, 1.5)	1.1 (0.7, 1.5)	1.1 (0.8, 1.3)	0.9 (0.7, 1.7)
B2 (mg)	1.2 (0.7, 1.6)	1.2 (0.7, 1.6)	1.2 (0.6, 1.7)	1.1 (0.7, 1.6)	1.3 (0.8, 2.1)
B3 (mg)	24.2 (17.6, 36.1)	25.7 (16.9, 38.2)	23.8 (20.0, 32.5)	25.9 (16.9, 38.2)	24.0 (19.4, 32.8)
B5 (mg)	3.5 (1.3, 4.4)	3.0 (1.3, 4.2)	3.7 (2.1, 5.6)	3.4 (1.6, 4.2)	3.7 (0.9, 4.9)
B6 (mg)	1.7 (1.0, 2.3)	1.65 (1.0, 2.3)	1.7 (0.9, 2.1)	1.9 (1.0, 2.2)	1.6 (1.0, 2.5)
B12 (mcg)	4.3 (1.0, 6.0)	4.1 (1.0, 5.3)	5.8 (1.7, 8.0)	3.5 (1.5, 5.9)	4.7 (0.8, 6.4)
C (mg)	65.2 (48.4, 132.7)	59.8 (43.1, 111.0)	112.7 (73.8, 170.8)	62.2 (47.7, 126.9)	79.5 (48.4, 132.7)
Ca (mg)	586.5 (365.8, 906.4)	619.9 (412.2, 906.4)	465.5 (292.5, 951.8)	496.0 (332.5, 690.1)	642.4 (417.2, 1138.8)
Folate (mcg)	234.0 (116.5, 303.4)	205.1 (116.5, 291.9)	268.9 (142.1, 324.0	203.2 (116.5, 293.7)	280.3 (119.4, 321.5)
Fe (mg)	14.0 (8.0, 18.4)	14.7 (8.6, 18.4)	10.9 (6.5, 18.2)	11.0 (7.2, 19.4)	15.5 (10.7, 18.4)
Na (mg)	4011.9 (2481.2, 5564.5)	4011.9 (2283.2, 5359.7)	4419.4 (3032.8, 5759.0)	3727.5 (2848.5, 5359.7)	4429.6 (2230.9, 6245.9)
Zinc (mg)	6.4 (3.1, 10.0)	5.8 (3.1, 10.0)	8.4 (3.3, 12.0)	5.8 (4.2, 9.0)	8.1 (1.4, 10.7)

Thai Dietary Reference Intake: vitamin A: 700 mcg RAE males ≥ 19 y, 600 mcg RAE females ≥ 19 y; B1: 1.2 mg males ≥ 13 y, 1.1 mg females ≥ 19 y; B2: 1.3 mg males ≥13 y, 1.1 mg females ≥ 19 y; B3: 16 mg males ≥ 13 y, 14 mg females ≥ 13 y; B5: 5 mg males, females ≥ 13 y; B6: 1.3 mg males 19-50 y, 1.7 mg males ≥ 51 y, 1.3 mg females 19-50 y, 1.5 mg females ≥ 51 y; B12: 2.4 mcg males, females ≥ 16 y; vitamin C: 100 mg males ≥ 19 y, 85 mg females ≥ 19 y; calcium 800 mcg males/females 19-50 y, 1000 mcg males/females ≥ 51y; folate: 300 mcg males/females ≥ 13 y; iron 11.5 mg males 19 60 y, 11 mg males ≥ 61 y, 20 mg females 19-50 y, 10 mg females ≥ 51 y; zinc: 11.6 mg males 19-30 y, 10.9 mg 31-70 y, 10.3 mg males ≥ 71 y, 9.7 mg females 19-30 y, 9.2 mg females 31-60; 8.6 mg females ≥ 61 y[24].

Table 3. Percentage (%) of participants meeting their daily requirements.

	Total (N=28)	Male (n=20)	Female (n=8)	Ration only (n=16)	Ration+ (n=12)
Calories	60.7	45.0	100.0[2]	56.3	66.7
Protein	53.6	45.0	75.0	43.8	66.7
Carbohydrates	89.3	95.0	75.0	93.8	83.3

For peer review only

Fats	60.7	60.0	62.5	56.3	66.7
A	3.6	0.0	12.5	6.3	0.0
B1	42.9	40.0	50.0	43.8	41.7
B2	53.6	55.0	50.0	50.0	58.3
B3	89.3	85.0	100.0	81.3	100.0
B5	17.9	10.0	37.5	12.5	25.0
B6	57.1	55.0	62.5	56.3	58.3
B12	64.3	60.0	75.0	62.5	66.7
C	42.9	30.0	75.0[3]	37.5	50.0
Ca	21.4	20.0	25.0	12.5	33.3
Folate	25.0	20.0	37.5	25.0	25.0
Fe	64.3	80.0[4]	25.0	62.5	66.7
Na[1]	25.0	30.0	12.5	18.8	33.3
Zinc	21.4	20.0	25.0	18.8	25.0

[1]Proportion of participants with sodium consumption below 2500 mg/24 h vs those with high sodium intake.
[2]Higher proportion of females meet caloric requirements than males (p=0.010, Fisher's exact test).
[3]Higher proportion of females meet vitamin C requirements than males (p=0.044, Fisher's exact test).
[4]Higher proportion of males meet iron intake requirements than females (p=0.011, Fisher's exact test).

For peer review only - http://bmjopen.bmj.com/site/about/guidelines.xhtml

Reporting checklist for cross sectional study.

Based on the STROBE cross sectional guidelines.

Instructions to authors

Complete this checklist by entering the page numbers from your manuscript where readers will find each of the items listed below.

Your article may not currently address all the items on the checklist. Please modify your text to include the missing information. If you are certain that an item does not apply, please write "n/a" and provide a short explanation.

Upload your completed checklist as an extra file when you submit to a journal.

In your methods section, say that you used the STROBE cross sectionalreporting guidelines, and cite them as:

von Elm E, Altman DG, Egger M, Pocock SJ, Gotzsche PC, Vandenbroucke JP. The Strengthening the Reporting of Observational Studies in Epidemiology (STROBE) Statement: guidelines for reporting observational studies.

For peer review only

		Reporting Item	Page Number
**Title and abstract**			
Title	#1a	Indicate the study’s design with a commonly used term in the title or the abstract	1
Abstract	#1b	Provide in the abstract an informative and balanced summary of what was done and what was found	2
**Introduction**			
Background / rationale	#2	Explain the scientific background and rationale for the investigation being reported	4
Objectives	#3	State specific objectives, including any prespecified hypotheses	4
**Methods**			
Study design	#4	Present key elements of study design early in the paper	5
Setting	#5	Describe the setting, locations, and relevant dates, including periods of	5

		recruitment, exposure, follow-up, and data collection	
Eligibility criteria	#6a	Give the eligibility criteria, and the sources and methods of selection of participants.	5
	#7	Clearly define all outcomes, exposures, predictors, potential confounders, and effect modifiers. Give diagnostic criteria, if applicable	6
Data sources / measurement	#8	For each variable of interest give sources of data and details of methods of assessment (measurement). Describe comparability of assessment methods if there is more than one group. Give information separately for for exposed and unexposed groups if applicable.	7
Bias	#9	Describe any efforts to address potential sources of bias	7
Study size	#10	Explain how the study size was arrived at	5
Quantitative variables	#11	Explain how quantitative variables were handled in the analyses. If applicable, describe which groupings were chosen, and why	8
Statistical methods	#12a	Describe all statistical methods, including those used to control for confounding	8
Statistical methods	#12b	Describe any methods used to examine subgroups and interactions	8
Statistical methods	#12c	Explain how missing data were addressed	NA
Statistical methods	#12d	If applicable, describe analytical methods taking account of sampling strategy	NA
Statistical methods	#12e	Describe any sensitivity analyses	NA
**Results**			
Participants	#13a	Report numbers of individuals at each stage of study—eg numbers potentially eligible, examined for eligibility, confirmed eligible, included in the study, completing follow-up, and analysed. Give information separately for for exposed and unexposed groups if applicable.	8
Participants	#13b	Give reasons for non-participation at each stage	NA
Participants	#13c	Consider use of a flow diagram	NA

For peer review only

Descriptive data	#14a	Give characteristics of study participants (eg demographic, clinical, social) and information on exposures and potential confounders. Give information separately for exposed and unexposed groups if applicable.	8
Descriptive data	#14b	Indicate number of participants with missing data for each variable of interest	NA
Outcome data	#15	Report numbers of outcome events or summary measures. Give information separately for exposed and unexposed groups if applicable.	9
Main results	#16a	Give unadjusted estimates and, if applicable, confounder-adjusted estimates and their precision (eg, 95% confidence interval). Make clear which confounders were adjusted for and why they were included	9
Main results	#16b	Report category boundaries when continuous variables were categorized	9
Main results	#16c	If relevant, consider translating estimates of relative risk into absolute risk for a meaningful time period	9
Other analyses	#17	Report other analyses done—e.g., analyses of subgroups and interactions, and sensitivity analyses	NA
**Discussion**			
Key results	#18	Summarise key results with reference to study objectives	9
Limitations	#19	Discuss limitations of the study, taking into account sources of potential bias or imprecision. Discuss both direction and magnitude of any potential bias.	11
Interpretation	#20	Give a cautious overall interpretation considering objectives, limitations, multiplicity of analyses, results from similar studies, and other relevant evidence.	11
Generalisability	#21	Discuss the generalisability (external validity) of the study results	11
**Other Information**			
Funding	#22	Give the source of funding and the role of the funders for the present study and, if applicable, for the original study on which the present article is based	12

The STROBE checklist is distributed under the terms of the Creative Commons Attribution License CC-BY.
This checklist was completed on 30. April 2021 using https://www.goodreports.org/, a tool made by the EQUATOR Network in collaboration with Penelope.ai

For peer review only

BMJ Open

A cross-sectional study of nutritional intake among patients undergoing tuberculosis treatment along the Myanmar-Thailand border

Journal:	*BMJ Open*
Manuscript ID	bmjopen-2021-052981.R1
Article Type:	Original research
Date Submitted by the Author:	12-Oct-2021
Complete List of Authors:	Damji, Karim; California State University Northridge<br>Hashmi, Ahmar; Chiang Mai University, Department of Family Medicine<br>Kyi, Lin Lin; Mahidol Oxford Tropical Medicine Research Unit, Shoklo Malaria Research Unit<br>Vincenti-Delmas, Michele; Mahidol Oxford Tropical Medicine Research Unit, Shoklo Malaria Research Unit<br>Htun, Win Pa Pa; Mahidol Oxford Tropical Medicine Research Unit, Shoklo Malaria Research Unit<br>Ko Ko Aung, Htet; Mahidol Oxford Tropical Medicine Research Unit, Shoklo Malaria Research Unit<br>Brummaier, Tobias; Mahidol Oxford Tropical Medicine Research Unit; Swiss Tropical and Public Health Institute<br>Angkurawaranon, Chaisiri; Faculty of Medicine, Chiang Mai University, Department of Family Medicine<br>Carrara, Verena; Mahidol Oxford Tropical Medicine Research Unit, Shoklo Malaria Research Unit<br>Nosten, Francois; Centre for Tropical Medicine and Global Health, Nuffield Department of Medicine, University of Oxford; Shoklo Malaria Research Unit, Mahidol-Oxford Tropical Medicine Research Unit, Faculty of Tropical Medicine, Mahidol University
**Primary Subject Heading**:	Nutrition and metabolism
Secondary Subject Heading:	Global health, Infectious diseases
Keywords:	Tuberculosis < INFECTIOUS DISEASES, NUTRITION & DIETETICS, EPIDEMIOLOGY, Tropical medicine < INFECTIOUS DISEASES

SCHOLARONE™
Manuscripts

I, the Submitting Author has the right to grant and does grant on behalf of all authors of the Work (as defined in the below author licence), an exclusive licence and/or a non-exclusive licence for contributions from authors who are: i) UK Crown employees; ii) where BMJ has agreed a CC-BY licence shall apply, and/or iii) in accordance with the terms applicable for US Federal Government officers or employees acting as part of their official duties; on a worldwide, perpetual, irrevocable, royalty-free basis to BMJ Publishing Group Ltd ("BMJ") its licensees and where the relevant Journal is co-owned by BMJ to the co-owners of the Journal, to publish the Work in this journal and any other BMJ products and to exploit all rights, as set out in our licence.

The Submitting Author accepts and understands that any supply made under these terms is made by BMJ to the Submitting Author unless you are acting as an employee on behalf of your employer or a postgraduate student of an affiliated institution which is paying any applicable article publishing charge ("APC") for Open Access articles. Where the Submitting Author wishes to make the Work available on an Open Access basis (and intends to pay the relevant APC), the terms of reuse of such Open Access shall be governed by a Creative Commons licence – details of these licences and which Creative Commons licence will apply to this Work are set out in our licence referred to above.

Other than as permitted in any relevant BMJ Author's Self Archiving Policies, I confirm this Work has not been accepted for publication elsewhere, is not being considered for publication elsewhere and does not duplicate material already published. I confirm all authors consent to publication of this Work and authorise the granting of this licence.

For peer review only

A cross-sectional study of nutritional intake among patients undergoing tuberculosis treatment along the Myanmar-Thailand border

Karim Damji[1], Ahmar H Hashmi[2]*, Lin Lin Kyi[3], Michele Vincenti-Delmas[3], Win Pa Pa Htun[3], Htet Ko Ko Aung[3], Tobias Brummaier[3,4,5,6], Chaisiri Angkurawaranon[2], Verena I Carrara[3,4,7], Francois Nosten[3,4]

[1]Family and Consumer Sciences, California State University, Northridge, USA

[2]Faculty of Medicine, Chiang Mai University, Chiang Mai, Thailand

[3]Shoklo Malaria Research Unit, Mahidol-Oxford Tropical Medicine Research Unit, Mae Sot, Thailand

[4] Centre for Tropical Medicine and Global Health, University of Oxford, Oxford, UK

[5]Swiss Tropical and Public Health Institute, Basel, Switzerland

[6]University of Basel, Basel, Switzerland

[7]Institute of Global Health, Faculty of Medicine, University of Geneva, Geneva, Switzerland

*Corresponding author: chaisiri.a@cmu.ac.th; Department of Family Medicine, Faculty of Medicine, Chiang Mai University, 110 Intharavoros Road, Chiang Mai 50220 Thailand

Karim Damji: karim.damji.945@my.csun.edu

Ahmar H Hashmi: ahmar.hashmi715@gmail.com

Lin Lin Kyi: nanlinlinkyi33@gmail.com

Michele Vincenti-Delmas: michele.vincenti@gmail.com

Win Pa Pa: winpapa@shoklo-unit.com

Htet Ko Ko Aung: htetkoko@shoklo-unit.com

Tobias Brummaier: tobias@shoklo-unit.com

Chaisiri Angkurawaranon: chaisiri.a@cmu.ac.th

Verena I Carrara: verena@shoklo-unit.com

Francois Nosten: francois@tropmedres.ac

For peer review only

For peer review only - http://bmjopen.bmj.com/site/about/guidelines.xhtml

Abstract

Objective: This study summarizes nutritional intake among TB patients along the Myanmar-Thailand border according to the local diet.

Setting: TB sanatorium along the Myanmar-Thailand border.

Participants: Cross-sectional surveys of 24-hour food recall were conducted with participants receiving anti-tuberculous treatment. Participants were purposively selected to reflect proportion of age, sex, and HIV co-infection based on historical patient records. Out of a total of 28 participants, 20 (71.4%) were male and 5 (17.9%) were co-infected with HIV.

Primary and secondary outcome measures: Primary outcome compared actual recorded intake and recommended intake. Secondary outcomes compared weight gain and BMI from diagnosis to time of survey.

Results: There were no significant differences in macro- or micronutrient intake when stratified by sex or for patients supplementing meal rations. Mean treatment length at time of survey was 20.7 weeks (95% CI: 16.5, 24.9). A significantly higher proportion of women (8/8, 100%) met caloric requirements compared to men (9/20, 45.0%, p=0.010), but few participants met other macro- or micronutrient requirements, with no significant differences by sex or for patients supplementing their rations. From diagnosis to the time of the survey, participants averaged significant weight gain of 6.48 kg (95% CI: 3.87, 9.10) and increased their BMI of 2.47 kg/m^2 (95% CI: 1.45, 3.49; p=0.0001 for both). However, 50% (14/28) still had mild or more severe forms of malnutrition.

Conclusions: This cross-sectional survey of nutritional intake in patients undergoing TB treatment in a sanatorium demonstrates the difficulty in sufficiently meeting nutritional demands, even when providing nutritional support.

Keywords: tuberculosis; malnutrition; nutrition assessment; nutritional support; transients and migrants.

Word count: 2619 words

For peer review only

For peer review only - http://bmjopen.bmj.com/site/about/guidelines.xhtml

Article Summary

Strengths and limitations of the study

- Strong methodology tailored to the local diet to accurately assess nutritional intake.
- Limited in sample size and not a controlled study of a nutritional intervention.
- Costlier measures that more precisely estimate nutritional intake were not possible in this study.

For peer review only

Introduction

The interaction of malnutrition and tuberculosis (TB) has been long established [1]. Not only does malnutrition predispose to TB infection, poor nutritional status and weight loss—particularly loss of lean body mass—are also associated with TB [2-4]. Malnutrition at the time of TB diagnosis increases mortality risk [2 5 6]. Inadequate nutritional intake is most likely the cause of poor nutritional status among patients with TB [1]. However, there is insufficient evidence to determine whether nutritional supplementation improves outcomes in patients with TB [3 7 8]. Even in situations where anti-tuberculous therapy and nutritional support is offered, many patients do not return to adequate nutritional status by the end of their treatment period [1 9]. But in light of the strong link between malnutrition and TB, the WHO insists on nutritional assessment, counseling, and addressing nutritional deficits for patients with TB [3 5 8 10 11].

However, it is difficult to assess nutritional intake of patients with TB to detect underlying nutritional deficits. Nutritional assessments commonly use 24-hour food recalls or food frequency questionnaires. In low- and middle-income countries—where the bulk of global TB cases occur—the lack of nutrient composition data for local diets leads to inaccurate estimates of nutrient intake [11]. These intake assessments relying on patient recall may be limited when anti-tuberculous treatment is provided as outpatient or community directly observed therapy (DOTS) programs [2 10].

Along the Myanmar-Thailand border, TB clinics provide services for migrants with TB via a unique, sanatorium model [12-14]. Nutritional support, in the way of rations, are included in the cadre of care and treatment services provided to patients who reside at the clinics for the duration of their treatment. Recent nutrition research has allowed a better characterization of the local diets along the border sufficient to determine nutritional content [15 16]. This cross-sectional study seeks to quantify macro- and micro-nutritional intake of a sample of patients

For peer review only

with TB along the border and to evaluate if meal rations adequately address patients' nutritional demands.

Methods

Setting: Tuberculosis Program along the border

Established in 2010, the Tuberculosis Program at the Borderlands Health Foundation/Shoklo Malaria Research Unit (SMRU) has provided a large breadth of services for TB patients through its sanatoria, including diagnostic and treatment procedures and also housing and accommodation, dietary, and psychosocial support services. By the end of January 2019, these clinics had provided treatment services to a total of 1925 patients, 1465 (76%) of whom had successfully completed the entirety of their treatment. At the time of this study, 118 patients were actively receiving treatment. Patients are agricultural migrants originally from either side of the Myanmar-Thailand border, predominantly of Burman or Karen ethnicity.

Study participants and design

Stratified, purposive sampling was performed to select patients from participating TB clinics. TB program patient records were reviewed to estimate the proportion of men and women, age, and those co-infected with HIV among the patient population. These proportions were then applied to the existing roster of patients currently under care and screened to determine eligibility. Participants included had TB confirmed by GeneXpert and/or AFB smear, with pulmonary or extrapulmonary infections, multi-drug resistant TB, or HIV co-infection. Routine treatment was provided to all patients according to WHO guidelines. Screened participants were excluded if they were in treatment for less than 8 weeks, those with recent acute onset of illness, those unable to provide accurate 24-hour food recalls, or those unable to provide consent.

Nutrition support provided

Patients are provided daily meals for the duration of their treatment at the sanatorium. Rations provided by the sanatorium include two full meals a day as well as refreshments

For peer review only

(processed foods and liquids). Some participants were able to supplement the rations provided with food from outside sources. In addition to daily meals, patients receive weekly rations which include biscuits, juice, and sweetened milk powders. While ingredients are budgeted for each patient, servings are not standardized. Each meal consisted of a serving of rice, and a curry. Protein sources for meals come from animal sources everyday out of the week, with a predominance of chicken and fish. The meals were provided in timing and frequency according to local eating habits. Chefs were locals from the communities, so were receptive to local dietary customs and preferences.

Survey tools and validation

24-hour food recalls were used to determine participant food intake, which was adapted from a form validated for use for an observational cohort, "Molecular signature of Karen and Burmese pregnant women on the Thailand-Myanmar border," conducted at SMRU (ClinicalTrials.gov Identifier: NCT02797327) [15]. However, as portion size estimations—using visual and other size estimation aids or household utensil measures—are limited [17-19], caloric intake is often underestimated, such as for cereal grains among other food items [19]. As a response, dietitians advocate the use of alternative methods including hand measurements to estimate portion sizes in 24-hour food recalls, which has been validated for accuracy and appropriateness of this method for use in low resource settings [15]. In previous 24-hour food recalls in migrant clinics along the Myanmar-Thailand border, patients often underestimated their intake of rice—similar to studies in Africa [19]. Therefore, 24-hour food recalls have employed all methods at our disposal: standardized measuring instruments, hand measurements, as well as visual aids [17]. Using multiple methods of portion size estimation may enhance the accuracy of recall data.

For peer review only

Calculating Nutritional Intake

Following survey completion, food recall data was uploaded into a, free online nutrition data software (Cronometer, https://cronometer.com/). Nutrition data was calculated for the total intake, intake from rations provided and intake excluding rice.

Recipes for all meals provided at the sanatorium as part of the rations were gathered through interviewing the cooks at the sanatorium and corroborating common recipes with several local staff at SMRU. This allowed compiling basic recipes for curries, soups, salads, stir fries, and pastes. Any dish or meal recalled as part of the rations allowed for the main ingredients to be determined and used in conjunction with basic recipes. These final recipes were then added to Cronometer.

As several ingredients in this community's diet were not available on Cronometer's database, nutrient data was clarified using the Mahidol University's Thai Food Composition Database or McGill University's Center for Indigenous Peoples' Nutrition and Environment Karen food composition database [20 21]. Nutritional information in these databases is often expressed in terms of nutrients per 100-gram sample, hence densities of ingredients was required. This was determined either through direct measurement, matching densities to foods with similar descriptions, or averaging the densities of similar ingredients.

Anthropometric measurements and determining nutritional requirements

Anthropometric measurements were performed for each participant immediately prior to conducting the 24-hour food recall and followed TB Program standard of procedures for weight and height assessment. Routinely calibrated mechanical scales were used to measure weight and height accurate to 500 grams and 5 mm, respectively. BMI was calculated and categorized into: severe malnutrition (< 16.0 kg/m^2), moderate malnutrition (16.0 – 16.9 kg/m^2), mild malnutrition (17.0 – 18.4 kg/m^2), normal (18.5 – 24.9 kg/m^2), and overweight (25.0 – 29.9 kg/m^2) [22].

For peer review only - http://bmjopen.bmj.com/site/about/guidelines.xhtml

Nutritional requirements for comparison against participants' actual intake were calculated for each individual patient. For TB patients without comorbidities, we used a protein range of 1.2-1.5 g/kg Ideal Body Weight (IBW). The energy range was 35-40 kcal/kg body weight. IBW was calculated using the Devine Equation (50kg +2.3kg X (Ht – 60 in) for men, and 45.5kg +2.3kg X (Ht - 60 in) for women), which was adapted for the metric system [23]. For patients with an HIV coinfection, the 35-40 kcal/kg range was increased by 20-30%. The estimated energy needs of the one participant with an above-the-knee amputation were reduced by 16%. National Academy of Medicine recommendations were used for carbohydrates and fats [24] and the Thai daily recommended intake (DRI) for micronutrients (vitamins A, B-complex, C, calcium, iron, sodium, zinc) [25].

Statistical Analysis

Analysis was conducted using Stata version 14 (StataCorp LP, College Station, TX, USA). Distributions of the data were assessed for normality, with descriptive data summarized using mean and 95% confidence intervals or median and inter-quartile range as appropriate. The primary outcome of this study was to compare participants' dietary intake with their individual nutritional requirements. Participant intake is summarized for all three macronutrients (protein, carbohydrates, and fats) and selected micronutrients. The secondary outcome assessed weight gain and change in BMI from enrollment in the TB sanatorium until the time of the survey. To allow for comparisons, these data were stratified according to sex and whether participants supplemented their rations with additional food. Statistical comparisons were then made using Student's t-test, Mann-Whitney U test, Wilcoxon signed rank test, or Chi-squared test according to continuous or categorical variables. Sex, age, BMI at diagnosis, and treatment length at time of survey were assessed for potential association with outcomes of interest.

For peer review only - http://bmjopen.bmj.com/site/about/guidelines.xhtml

Patient and public involvement in research

This study was initially begun as an audit of nutrition services provided at the TB clinic to improve patient diet. Although patients were satisfied overall, we attempted to understand if nutrition provided actual met their required needs. Study findings will be disseminated to both patients and staff at the clinics. In addition, the research team engaged the local population through a local community advisory board, the Tak Community Advisory Board, Thailand. Comprised of community leaders, they advised on study design and implementation and approved the study (TCAB 201903).

Results

Table 1 highlights basic participants' information for all 28 participants. More men were included in the analysis (20/28, 71.4% vs 8/28, 28.6%) and were significantly older than women (52.4 vs 37.6 years, p=0.021). A total of 17.9% (5/28) of participants were coinfected with HIV. None of the participants reported substance use such as alcohol, cigarettes, or other drugs. Just over half the participants (57.1%, 16/28) ate only the rations provided by the clinic. No participants had MDRTB, and only one male participant (1/20, 5%) had an extrapulmonary form of TB. At diagnosis, men were significantly taller (160.7 vs 150.8 cm, p=0.002) and heavier (43.2 vs 36.9 kg, p=0.011) than women, but there were no significant differences for BMI (16.7 vs 16.3 kg/m^2, p=630). All participants were currently receiving treatment with a mean treatment length at the time of survey of 20.7 weeks (95% CI: 16.5-24.8). Pairwise comparisons show 22/28 (78.6%) participants had increased weight and BMI from diagnosis to the time of the survey; with an average gain of 6.48 kg (95% CI: 3.87, 9.10) and 2.47 kg/m^2 (95% CI: 1.45, 3.49) per participant (p=0.0001 for both). By the time of the survey, weight of men and women were no longer significantly different (51.0 vs 44.0 kg, p=0.115). This improvement in weight and BMI is reflected in the shift of malnutrition category from diagnosis to the time of survey: at the time of diagnosis 89% (25/28) of

For peer review only - http://bmjopen.bmj.com/site/about/guidelines.xhtml

participants had mild or more severe forms of malnutrition compared to 50% (14/28) of participants at the time of the survey (p=0.22).

Table 2 outlines the average nutritional intake stratified by sex and whether participants ate rations only. There were no significant differences in total nutritional intake for macro- or micronutrient when comparing men to women, or patients eating only the rations provided compared to those supplementing the rations received.

Table 3 highlights the proportion of participants meeting their recommended daily allowances. Participants' diets were low in protein, fats, and nearly all vitamins, but were high in salt content. A significantly higher proportion of women met their overall caloric and vitamin C requirements (p=0.010, p=0.044 respectively), while a higher proportion of men met their iron intake requirements (p=0.011). Meeting the remaining dietary requirements did not differ significantly between the sexes or having a ration-only diet.

Discussion

This study presents findings from a cross-sectional survey of TB patients undergoing treatment while residing at a sanatorium along the Myanmar-Thailand border. The most notable finding in this study is the difficulty patients had reaching their nutrition requirements. Even TB patients supplementing the rations provided by the clinic were not more likely to meet their requirements than those eating only the provided rations. Total calories and protein requirements were often not met, and although carbohydrates approached the needed requirements, that most of the total carbohydrates were acquired from rice reflects a lack of dietary diversity. This lack of dietary diversity likely relates to participants' being unable to meet most micronutrient requirements. Similar to other study findings, although there was some weight gain and improvement in BMI, and malnutrition status, this was not to the level desired [1 8 9]. Overall intake of almost all important micronutrients was limited, with

For peer review only - http://bmjopen.bmj.com/site/about/guidelines.xhtml

sodium intake often being too high—even for patients eating only the rations provided. This is most likely due to the limited diversity represented in the patients' diets.

These inadequacies in nutrient intake among TB patients echoes findings in other studies [1 2 7 9 26]. This study adds to the literature by including robust data on the nutritional content of local diets; provides strong nutritional intake data given the sanatorium setting; and provides baseline data for a vulnerable, migrant population that likely has worse nutritional status upon diagnosis than may be seen in other populations. Compared to other settings, where nutrient intake was estimated in accordance to the local diet, TB patients in Georgia were able to manage high caloric intake for follow-up in an outpatient setting [10].

In this setting in Asia, it may be useful to highlight some recommendations for increasing nutritional intake: a regular source of protein (animal-based if possible); small yet regular meals; and locally sourced fresh fruits and vegetables or other snacks low in sodium and saturated fats that provide additional micronutrient supplementation. These recommendations should be considered alongside regular dietary counseling and support—if human resources can be allocated—as recommended by the WHO [8]. Not only are patients recommended to have an assessment when enrolled for care but support may also be important at the beginning stages of drug treatment, when side effects such as nausea, vomiting, and loss of appetite can interfere with early nutritional gains [26]. A culturally-tailored counseling program, representative of the local diet, can help inform TB patients about the importance of a diverse diet, especially when undergoing treatment and as local community awareness about good nutrition may be limited. Including family members in these discussions would be helpful as family members often stay with patients in the sanatoria. Such counseling can also help inform TB patients (and their caregivers) of good nutritional foods should they be able to purchase foods to supplement their rations.

For peer review only

This study is not without limitations. It is cross-sectional in design—although this is the design reported by most studies of this nature. With further funding, future studies will assess nutritional intake in addition to blood and serum measures of nutritional status among a larger sample of patients. The comparisons drawn in this sample are limited as well, given the broad inclusion criteria (e.g., including HIV co-infected) that were chosen to include a more representative sample of patients from this border region to inform future nutritional interventions. Future studies may also benefit from using additional anthropometric measures, especially those more sensitive in detecting undernutrition [6 9 27 28] and that account for disease severity [2 5]. Some limitation is inherent to the methods used such as not performing serial food recall surveys, measurement intake accuracy, and recall bias[17 18 29], and aligning food descriptions from the local diet with food composition databases, but the tools used have been developed across different nutritional studies in this area and have allowed for validating methods across different segments of the local migrant populations.

Conclusions

This cross-sectional survey of TB patients residing in a sanatorium along the Myanmar-Thailand border demonstrates the difficulty in sufficiently meeting nutritional demands, even when providing nutritional support.

Abbreviations

BMI	Body-mass index
DRI	Daily recommended intake
SMRU	Shoklo Malaria Research Unit
TB	Tuberculosis
WHO	World Health Organization

Acknowledgements

We are grateful for the patients who volunteered to be a part of this study. We would like to acknowledge the staff working in the TB department at SMRU, for their help and support with logistics, materials, and other needs. We would like to thank the local SMRU staff that

For peer review only

helped with recipes and understanding local diets, including the cooks at the TB sanatorium. We would like to thank Wolfgang Stuetz for feedback on drafts of this manuscript.

Ethics approval

This study received ethical approval from the Research Ethics Committee of the Faculty of Medicine, Chiang Mai University (FAM-2562-06197) in accordance with the Declaration of Helsinki. Informed verbal assent was given by all participants prior to survey, with written informed consent waived by the Research Ethics Committee of Chiang Mai University.

Consent for publication

Not applicable.

Data availability statement

The datasets generated and/or analyzed during the current study are not publicly available due to privacy/safety for migrant participants, but are available from the corresponding author on reasonable request.

Competing interests

The authors declare that they have no competing interests.

Funding

This research was funded in whole, or in part, by the Wellcome Trust Thailand/Laos Major Overseas Program Renewal [grant number 106698]. For the purpose of open access, the author has applied a CC BY public copyright licence to any Author Accepted Manuscript version arising from this submission. The funding body had no role in the design of the study and collection, analysis, and interpretation of data and in writing the manuscript.

Author contributions

KD, AHH, MVD, CA, VIC, FN: study conception, design; KD, AHH, LLK, WPPH, HKKA, TB: data collection and study implementation; KD, AHH, VIC: data analysis/interpretation; All authors critically revised the manuscript and approved the final version submitted and agree to be accountable for this work.

For peer review only

References

1. Macallan DC. Malnutrition in tuberculosis. *Diagnostic microbiology and infectious disease* 1999;34(2):153-7.
2. Mupere E, Parraga IM, Tisch DJ, et al. Low nutrient intake among adult women and patients with severe tuberculosis disease in Uganda: a cross-sectional study. *BMC Public Health* 2012;12:1050.
3. Sinclair D, Abba K, Grobler L, et al. Nutritional supplements for people being treated for active tuberculosis. *Cochrane Database of Systematic Reviews* 2011;11:CD006086.
4. White LV, Edwards T, Lee N, et al. Patterns and predictors of co-morbidities in Tuberculosis: A cross-sectional study in the Philippines. *Scientific Reports* 2020;10:4100.
5. Miyata S, Tanaka M, Ihaku D. The prognostic significance of nutritional status using malnutrition universal screening tool in patients with pulmonary tuberculosis. *Nutr J* 2013;12:42.
6. Zachariah R, Spielmann MP, Harries AD, et al. Moderate to severe malnutrition in patients with tuberculosis is a risk factor associated with early death. *Transactions of the Royal Society of Tropical Medicine and Hygiene* 2002;96(3):291-4.
7. Rudolph M, Kroll F, Beery M, et al. A pilot study assessing the impact of a fortified supplementary food on the health and well-being of creche children and adult TB patients in South Africa. *PLoS ONE [Electronic Resource]* 2013;8(1):e55544.
8. World Health Organization. Nutritional care and support for patients with tuberculosis. Geneva: WHO, 2013.
9. Bacelo AC, Ramalho A, Brasil PE, et al. Nutritional supplementation is a necessary complement to dietary counseling among tuberculosis and tuberculosis-HIV patients. *PloS one* 2015;10(8):e0134785.
10. Frediani JK, Sanikidze E, Kipiani M, et al. Macronutrient intake and body composition changes during anti-tuberculosis therapy in adults. *Clin Nutr* 2016;35(1):205-12.
11. Frediani JK, Tukvadze N, Sanikidze E, et al. A culture-specific nutrient intake assessment instrument in patients with pulmonary tuberculosis. *Clin Nutr* 2013;32(6):1023-8.
12. Thi SS, Parker DM, Swe LL, et al. Migration histories of multidrug-resistant tuberculosis patients from the Thailand-Myanmar border, 2012-2014. *Int J Tuberc Lung Dis* 2017;21(7):753-58.
13. Tschirhart N, Nosten F, Foster AM. Migrant tuberculosis patient needs and health system response along the Thailand-Myanmar border. *Health Policy Planning* 2017;32:1212-19.
14. Tschirhart N, Sein T, Nosten F, et al. Migrant and refugee patient perspectives on travel and tuberculosis along the Thailand-Myanmar border: a qualitative study. *PLOS ONE* 2016;11(8):e0160222.
15. Brummaier T, Kabeer BSA, Lindow S, et al. A prospective cohort for the investigation of alteration in temporal transcriptional and microbiome trajectories preceding preterm birth: a study protocol. *BMJ Open* 2018;9:e023417.
16. Brummaier T, Kabeer BSA, Wilaisrisak P, et al. Cohort profile: molecular signature in pregnancy (MSP): longitudinal high-frequency sampling to characterise cross-omic trajectories in pregnancy in a resource-constrained setting. *BMJ Open* 2020;10:e041631.
17. Amoutzopoulos B, Paige P, Roberts C, et al. Portion size estimation in dietary assessment: a systematic review of existing tools, their strengths and limitations. *Nutrition Reviews* 2020;78(11)
18. Gibson AA, Hsu MS, Rangan AM, et al. Accuracy of hands *v.* household measures as portion size estimation aids. *J Nutritional Sci* 2016;5(e29)

For peer review only - http://bmjopen.bmj.com/site/about/guidelines.xhtml

19. Ogunbode AM, Owolabi MO, Ogunbode OO, et al. What's in your hands? A systematic review of dietary assessment methods and estimation of food sizes in a primary care clinic. *J Med Tropics* 2018;20(2):93.
20. Mahidol University, Institute of Nutrition. Thai Food Composition Database 2015 [Available from: http://www.inmu.mahidol.ac.th/thaifcd.
21. McGill University, Environment CfIPNa. CINE Global Health Case Study - Karen 2019 [Available from: https://www.mcgill.ca/cine/resources/data/karen.
22. World Health Organization. Management of severe malnutrition: a manual for physicians and other senior health workers. Geneva: WHO, 1999.
23. Peterson CM, Thomas DM, Blackburn GL, et al. Universal equation for estimating ideal body weight and body weight at any BMI. *Am J Clin Nutr* 2016;103(5):1197-203.
24. National Academy of Medicine. Dietary reference intakes for energy, carbohydrate, fiber, fat, fatty acids, cholesterol, protein, and amino acids. Washington, D.C.: National Academy of Medicine, 2005.
25. Bureau of Nutrition, Department of Health, Thailand Ministry of Public Health. Dietary reference intake for Thais 2020. Bangkok: Ministry of Public Health, 2020.
26. Bacelo AC, do Brasil PE, Cople-Rodrigues CD, et al. Dietary counseling adherence during tuberculosis treatment: A longitudinal study. *Clin Nutr ESPEN* 2017;17:44-53.
27. Lee N, White LV, Marin FP, et al. Mid-upper arm circumference predicts death in adult patients admitted to a TB ward in the Philippines: a prospective cohort study. *PLOS ONE* 2019;14(6):e0218193.
28. White LV, Lee N, Marin FP, et al. Performance of alternative measures to body mass index in the assessment of moderate and severe under-nutrition among acutely unwell patients hospitalized in a TB ward in the Philippines: a cross-sectional study. *PLOS ONE* 2019;14(5):e0215968.
29. Péter S, Saris WHM, Mathers J, et al. Nutrient status assessment in individuals and populations for healthy aging—statement from an expert workshop. *Nutrients* 2015;7(12):10491-500.

For peer review only

For peer review only - http://bmjopen.bmj.com/site/about/guidelines.xhtml

382 Table 1. Basic demographic, treatment, and anthropometric variables.

	All participants (N=28)	Male[1] (20, 71.4%)	Female (8 (28.6%)	
Variables				
Age (mean, 95% CI, years)	48.2 (42.1, 54.2)	52.4 (45.9, 58.9)	37.6 (24.8, 50.5)	p=0.021
Ration only (n, %)				p=0.69
Yes	16 (57.1)	12 (60.0)	4 (50.0)	
No	12 (42.9)	8 (40.0)	4 (50.0)	
HIV coinfection (n, %)				p=1.00
Positive	5 (17.9)	4 (20.0)	1 (12.5)	
Negative	23 (82.1)	16 (80.0)	7 (87.2)	
Treatment length at time of survey (mean, 95% CI, weeks)	20.7 (16.5, 24.8)	20.1 (15.4, 24.8)	22.2 (11.4, 32.9)	p=0.628
Height (mean, 95% CI, cm)	157.8 (154.6, 161.1)	160.7 (157.4, 163.9)	150.8 (144.4, 157.2)	p=0.003
Weight at diagnosis (mean, 95% CI, kg)	41.4 (39.1, 43.8)	43.2 (40.7, 45.8)	36.9 (31.9, 41.8)	p=0.011
Weight at survey (mean, 95% CI, kg)	49.0 (44.9, 53.0)	51.0 (46.3, 55.6)	44.0 (35.1, 52.9)	p=0.115
BMI at diagnosis (mean, 95% CI, kg/m^2)	16.1 (15.7, 17.5)	16.7 (15.7, 17.8)	16.3 (13.8, 18.7)	p=0.630
BMI at survey (mean, 95% CI, kg/m^2)	19.5 (18.3, 20.7)	19.7 (18.3, 21.1)	19.1 (16.3, 21.9)	p=0.682

383 [1]Includes one participant with an amputation of one leg and one with extrapulmonary TB.

384

385 Table 2. Nutrition intake reported as median (IQR).

	Totals (N=28)	Male (n=20)	Female (n=8)	Ration only (n=16)	Ration+ (n=12)
Calories (kcal)	2153.5 (1764.0, 3118.0)	2065.0 (1487.5, 3118.0)	2325.0 (1986.5, 3158.5)	2035.0 (1454.5, 2721.5)	2153.5 (1968.0, 3638.0)
Calories from rice (kcal)	967.0 (789.0, 1396.5)	967.0 (842.0, 1450.5)	967.0 (698.5, 1396.5)	967.0 (644.5, 1397.0)	967.5 (789.0, 1396.5)
Protein (kcal)	277.6 (192.6, 371.6)	268.6 (178.4, 371.6)	305.8 (225.8, 421.4)	268.6 (163.6, 360.8)	302.4 (225.8, 422.0)
Carbohydrates (kcal)	1240.4 (1061.6, 1718.0)	1248.0 (1025.6, 1891.0)	1231.8 (1098.0, 1522.0)	1160.2 (1008.6, 1718.0)	1313.8 (1173.8, 1791.8)
Fats (kcal)	540.9 (385.7, 797.4)	455.9 (383.9, 736.7)	756.9 (526.5, 982.4)	464.9 (356.4, 711.0)	736.7 (403.2, 947.7)

For peer review only - http://bmjopen.bmj.com/site/about/guidelines.xhtml

Vitamin A (mcg RAE)	293.8 (150.5, 459.4)	226.8 (150.5, 459.4)	360.7 (150.6, 494.2)	226.8 (1510.5, 425.6)	398.8 (141.9, 502.6)
Vitamin B1 (mg)	1.0 (0.7, 1.5)	1.0 (0.8, 1.5)	1.1 (0.7, 1.5)	1.1 (0.8, 1.3)	0.9 (0.7, 1.7)
Vitamin B2 (mg)	1.2 (0.7, 1.6)	1.2 (0.7, 1.6)	1.2 (0.6, 1.7)	1.1 (0.7, 1.6)	1.3 (0.8, 2.1)
Vitamin B3 (mg)	24.2 (17.6, 36.1)	25.7 (16.9, 38.2)	23.8 (20.0, 32.5)	25.9 (16.9, 38.2)	24.0 (19.4, 32.8)
Vitamin B5 (mg)	3.5 (1.3, 4.4)	3.0 (1.3, 4.2)	3.7 (2.1, 5.6)	3.4 (1.6, 4.2)	3.7 (0.9, 4.9)
Vitamin B6 (mg)	1.7 (1.0, 2.3)	1.65 (1.0, 2.3)	1.7 (0.9, 2.1)	1.9 (1.0, 2.2)	1.6 (1.0, 2.5)
Vitamin B9 (mcg)	234.0 (116.5, 303.4)	205.1 (116.5, 291.9)	268.9 (142.1, 324.0	203.2 (116.5, 293.7)	280.3 (119.4, 321.5)
Vitamin B12 (mcg)	4.3 (1.0, 6.0)	4.1 (1.0, 5.3)	5.8 (1.7, 8.0)	3.5 (1.5, 5.9)	4.7 (0.8, 6.4)
Vitamin C (mg)	65.2 (48.4, 132.7)	59.8 (43.1, 111.0)	112.7 (73.8, 170.8)	62.2 (47.7, 126.9)	79.5 (48.4, 132.7)
Calcium (mg)	586.5 (365.8, 906.4)	619.9 (412.2, 906.4)	465.5 (292.5, 951.8)	496.0 (332.5, 690.1)	642.4 (417.2, 1138.8)
Iron (mg)	14.0 (8.0, 18.4)	14.7 (8.6, 18.4)	10.9 (6.5, 18.2)	11.0 (7.2, 19.4)	15.5 (10.7, 18.4)
Sodium (mg)	4011.9 (2481.2, 5564.5)	4011.9 (2283.2, 5359.7)	4419.4 (3032.8, 5759.0)	3727.5 (2848.5, 5359.7)	4429.6 (2230.9, 6245.9)
Zinc (mg)	6.4 (3.1, 10.0)	5.8 (3.1, 10.0)	8.4 (3.3, 12.0)	5.8 (4.2, 9.0)	8.1 (1.4, 10.7)

Thai Dietary Reference Intake: vitamin A: 700 mcg RAE males ≥ 19 y, 600 mcg RAE females ≥ 19 y; B1: 1.2 mg males ≥ 13 y, 1.1 mg females ≥ 19 y; B2: 1.3 mg males ≥13 y, 1.1 mg females ≥ 19 y; B3: 16 mg males ≥ 13 y, 14 mg females ≥ 13 y; B5: 5 mg males, females ≥ 13 y; B6: 1.3 mg males 19-50 y, 1.7 mg males ≥ 51 y, 1.3 mg females 19-50 y, 1.5 mg females ≥ 51 y; B12: 2.4 mcg males, females ≥ 16 y; vitamin C: 100 mg males ≥ 19 y, 85 mg females ≥ 19 y; calcium 800 mcg males/females 19-50 y, 1000 mcg males/females ≥ 51y; folate: 300 mcg males/females ≥ 13 y; iron 11.5 mg males 19 60 y, 11 mg males ≥ 61 y, 20 mg females 19-50 y, 10 mg females ≥ 51 y; sodium: all participants require less than 2500 mg per day; zinc: 11.6 mg males 19-30 y, 10.9 mg 31-70 y, 10.3 mg males ≥ 71 y, 9.7 mg females 19-30 y, 9.2 mg females 31-60; 8.6 mg females ≥ 61 y [24].

Table 3. Percentage (%) of participants meeting their daily requirements.

	Total (N=28)	Male (n=20)	Female (n=8)	Ration only (n=16)	Ration+ (n=12)
Calories	60.7	45.0	100.0[2]	56.3	66.7
Protein	53.6	45.0	75.0	43.8	66.7
Carbohydrates	89.3	95.0	75.0	93.8	83.3

For peer review only - http://bmjopen.bmj.com/site/about/guidelines.xhtml

Fats	60.7	60.0	62.5	56.3	66.7
Vitamin A	3.6	0.0	12.5	6.3	0.0
Vitamin B1	42.9	40.0	50.0	43.8	41.7
Vitamin B2	53.6	55.0	50.0	50.0	58.3
Vitamin B3	89.3	85.0	100.0	81.3	100.0
Vitamin B5	17.9	10.0	37.5	12.5	25.0
Vitamin B6	57.1	55.0	62.5	56.3	58.3
Vitamin B9	25.0	20.0	37.5	25.0	25.0
Vitamin B12	64.3	60.0	75.0	62.5	66.7
Vitamin C	42.9	30.0	75.0[3]	37.5	50.0
Calcium	21.4	20.0	25.0	12.5	33.3
Iron	64.3	80.0[4]	25.0	62.5	66.7
Sodium[1]	25.0	30.0	12.5	18.8	33.3
Zinc	21.4	20.0	25.0	18.8	25.0

[1]Proportion of participants with sodium consumption below 2500 mg/24 h vs those with high sodium intake.
[2]Higher proportion of females met caloric requirements than males (p=0.010, Fisher's exact test).
[3]Higher proportion of females met vitamin C requirements than males (p=0.044, Fisher's exact test).
[4]Higher proportion of males met iron intake requirements than females (p=0.011, Fisher's exact test).

For peer review only

Reporting checklist for cross sectional study.

Based on the STROBE cross sectional guidelines.

Instructions to authors

Complete this checklist by entering the page numbers from your manuscript where readers will find each of the items listed below.

Your article may not currently address all the items on the checklist. Please modify your text to include the missing information. If you are certain that an item does not apply, please write "n/a" and provide a short explanation.

Upload your completed checklist as an extra file when you submit to a journal.

In your methods section, say that you used the STROBE cross sectionalreporting guidelines, and cite them as:

von Elm E, Altman DG, Egger M, Pocock SJ, Gotzsche PC, Vandenbroucke JP. The Strengthening the Reporting of Observational Studies in Epidemiology (STROBE) Statement: guidelines for reporting observational studies.

For peer review only

		Reporting Item	Page Number
**Title and abstract**			
Title	#1a	Indicate the study’s design with a commonly used term in the title or the abstract	1
Abstract	#1b	Provide in the abstract an informative and balanced summary of what was done and what was found	2
**Introduction**			
Background / rationale	#2	Explain the scientific background and rationale for the investigation being reported	4
Objectives	#3	State specific objectives, including any prespecified hypotheses	4
**Methods**			
Study design	#4	Present key elements of study design early in the paper	5
Setting	#5	Describe the setting, locations, and relevant dates, including periods of	5

		recruitment, exposure, follow-up, and data collection	
Eligibility criteria	#6a	Give the eligibility criteria, and the sources and methods of selection of participants.	5
	#7	Clearly define all outcomes, exposures, predictors, potential confounders, and effect modifiers. Give diagnostic criteria, if applicable	6
Data sources / measurement	#8	For each variable of interest give sources of data and details of methods of assessment (measurement). Describe comparability of assessment methods if there is more than one group. Give information separately for for exposed and unexposed groups if applicable.	7
Bias	#9	Describe any efforts to address potential sources of bias	7
Study size	#10	Explain how the study size was arrived at	5
Quantitative variables	#11	Explain how quantitative variables were handled in the analyses. If applicable, describe which groupings were chosen, and why	8
Statistical methods	#12a	Describe all statistical methods, including those used to control for confounding	8
Statistical methods	#12b	Describe any methods used to examine subgroups and interactions	8
Statistical methods	#12c	Explain how missing data were addressed	NA
Statistical methods	#12d	If applicable, describe analytical methods taking account of sampling strategy	NA
Statistical methods	#12e	Describe any sensitivity analyses	NA
**Results**			
Participants	#13a	Report numbers of individuals at each stage of study—eg numbers potentially eligible, examined for eligibility, confirmed eligible, included in the study, completing follow-up, and analysed. Give information separately for for exposed and unexposed groups if applicable.	8
Participants	#13b	Give reasons for non-participation at each stage	NA
Participants	#13c	Consider use of a flow diagram	NA

For peer review only

Descriptive data	#14a	Give characteristics of study participants (eg demographic, clinical, social) and information on exposures and potential confounders. Give information separately for exposed and unexposed groups if applicable.	8
Descriptive data	#14b	Indicate number of participants with missing data for each variable of interest	NA
Outcome data	#15	Report numbers of outcome events or summary measures. Give information separately for exposed and unexposed groups if applicable.	9
Main results	#16a	Give unadjusted estimates and, if applicable, confounder-adjusted estimates and their precision (eg, 95% confidence interval). Make clear which confounders were adjusted for and why they were included	9
Main results	#16b	Report category boundaries when continuous variables were categorized	9
Main results	#16c	If relevant, consider translating estimates of relative risk into absolute risk for a meaningful time period	9
Other analyses	#17	Report other analyses done—e.g., analyses of subgroups and interactions, and sensitivity analyses	NA
**Discussion**			
Key results	#18	Summarise key results with reference to study objectives	9
Limitations	#19	Discuss limitations of the study, taking into account sources of potential bias or imprecision. Discuss both direction and magnitude of any potential bias.	11
Interpretation	#20	Give a cautious overall interpretation considering objectives, limitations, multiplicity of analyses, results from similar studies, and other relevant evidence.	11
Generalisability	#21	Discuss the generalisability (external validity) of the study results	11
**Other Information**			
Funding	#22	Give the source of funding and the role of the funders for the present study and, if applicable, for the original study on which the present article is based	12

The STROBE checklist is distributed under the terms of the Creative Commons Attribution License CC-BY. This checklist was completed on 30. April 2021 using https://www.goodreports.org/, a tool made by the EQUATOR Network in collaboration with Penelope.ai

For peer review only

BMJ Open

A cross-sectional study of nutritional intake among patients undergoing tuberculosis treatment along the Myanmar-Thailand border

Journal:	*BMJ Open*
Manuscript ID	bmjopen-2021-052981.R2
Article Type:	Original research
Date Submitted by the Author:	17-Dec-2021
Complete List of Authors:	Damji, Karim; California State University Northridge<br>Hashmi, Ahmar; Chiang Mai University, Department of Family Medicine<br>Kyi, Lin Lin; Mahidol Oxford Tropical Medicine Research Unit, Shoklo Malaria Research Unit<br>Vincenti-Delmas, Michele; Mahidol Oxford Tropical Medicine Research Unit, Shoklo Malaria Research Unit<br>Htun, Win Pa Pa; Mahidol Oxford Tropical Medicine Research Unit, Shoklo Malaria Research Unit<br>Ko Ko Aung, Htet; Mahidol Oxford Tropical Medicine Research Unit, Shoklo Malaria Research Unit<br>Brummaier, Tobias; Mahidol Oxford Tropical Medicine Research Unit; Swiss Tropical and Public Health Institute<br>Angkurawaranon, Chaisiri; Faculty of Medicine, Chiang Mai University, Department of Family Medicine<br>Carrara, Verena; Mahidol Oxford Tropical Medicine Research Unit, Shoklo Malaria Research Unit<br>Nosten, Francois; Centre for Tropical Medicine and Global Health, Nuffield Department of Medicine, University of Oxford; Shoklo Malaria Research Unit, Mahidol-Oxford Tropical Medicine Research Unit, Faculty of Tropical Medicine, Mahidol University
**Primary Subject Heading**:	Nutrition and metabolism
Secondary Subject Heading:	Global health, Infectious diseases
Keywords:	Tuberculosis < INFECTIOUS DISEASES, NUTRITION & DIETETICS, EPIDEMIOLOGY, Tropical medicine < INFECTIOUS DISEASES

SCHOLARONE™
Manuscripts

I, the Submitting Author has the right to grant and does grant on behalf of all authors of the Work (as defined in the below author licence), an exclusive licence and/or a non-exclusive licence for contributions from authors who are: i) UK Crown employees; ii) where BMJ has agreed a CC-BY licence shall apply, and/or iii) in accordance with the terms applicable for US Federal Government officers or employees acting as part of their official duties; on a worldwide, perpetual, irrevocable, royalty-free basis to BMJ Publishing Group Ltd ("BMJ") its licensees and where the relevant Journal is co-owned by BMJ to the co-owners of the Journal, to publish the Work in this journal and any other BMJ products and to exploit all rights, as set out in our licence.

The Submitting Author accepts and understands that any supply made under these terms is made by BMJ to the Submitting Author unless you are acting as an employee on behalf of your employer or a postgraduate student of an affiliated institution which is paying any applicable article publishing charge ("APC") for Open Access articles. Where the Submitting Author wishes to make the Work available on an Open Access basis (and intends to pay the relevant APC), the terms of reuse of such Open Access shall be governed by a Creative Commons licence – details of these licences and which Creative Commons licence will apply to this Work are set out in our licence referred to above.

Other than as permitted in any relevant BMJ Author's Self Archiving Policies, I confirm this Work has not been accepted for publication elsewhere, is not being considered for publication elsewhere and does not duplicate material already published. I confirm all authors consent to publication of this Work and authorise the granting of this licence.

For peer review only

A cross-sectional study of nutritional intake among patients undergoing tuberculosis treatment along the Myanmar-Thailand border

Karim Damji[1], Ahmar H Hashmi[2]*, Lin Lin Kyi[3], Michele Vincenti-Delmas[3], Win Pa Pa Htun[3], Htet Ko Ko Aung[3], Tobias Brummaier[3,4,5,6], Chaisiri Angkurawaranon[2], Verena I Carrara[3,4,7], Francois Nosten[3,4]

[1]Family and Consumer Sciences, California State University, Northridge, USA

[2]Faculty of Medicine, Chiang Mai University, Chiang Mai, Thailand

[3]Shoklo Malaria Research Unit, Mahidol-Oxford Tropical Medicine Research Unit, Mae Sot, Thailand

[4] Centre for Tropical Medicine and Global Health, University of Oxford, Oxford, UK

[5]Swiss Tropical and Public Health Institute, Basel, Switzerland

[6]University of Basel, Basel, Switzerland

[7]Institute of Global Health, Faculty of Medicine, University of Geneva, Geneva, Switzerland

*Corresponding author: chaisiri.a@cmu.ac.th; Department of Family Medicine, Faculty of Medicine, Chiang Mai University, 110 Intharavoros Road, Chiang Mai 50220 Thailand

Karim Damji: karim.damji.945@my.csun.edu

Ahmar H Hashmi: ahmar.hashmi715@gmail.com

Lin Lin Kyi: nanlinlinkyi33@gmail.com

Michele Vincenti-Delmas: michele.vincenti@gmail.com

Win Pa Pa: winpapa@shoklo-unit.com

Htet Ko Ko Aung: htetkoko@shoklo-unit.com

Tobias Brummaier: tobias@shoklo-unit.com

Chaisiri Angkurawaranon: chaisiri.a@cmu.ac.th

Verena I Carrara: verena@shoklo-unit.com

Francois Nosten: francois@tropmedres.ac

For peer review only

Abstract

Objective: This study summarizes nutritional intake among TB patients along the Myanmar-Thailand border according to the local diet.

Setting: TB clinic along the Myanmar-Thailand border.

Participants: Cross-sectional surveys of 24-hour food recall were conducted with participants receiving anti-tuberculous treatment. Participants were purposively selected to reflect proportion of age, sex, and HIV co-infection based on historical patient records. Out of a total of 28 participants, 20 (71.4%) were male and 5 (17.9%) were co-infected with HIV.

Primary and secondary outcome measures: The primary outcome compared actual recorded intake and recommended intake. Secondary outcomes compared weight gain and BMI from diagnosis to time of survey.

Results: There were no significant differences in macro- or micronutrient intake when stratified by sex or for patients supplementing their rations. Mean treatment length at time of survey was 20.7 weeks (95% CI: 16.5, 24.9). A significantly higher proportion of women (8/8, 100%) met caloric requirements compared to men (9/20, 45.0%, p=0.010), but few participants met other macro- or micronutrient requirements, with no significant differences by sex or for patients supplementing their rations. From diagnosis to the time of the survey, participants averaged significant weight gain of 6.48 kg (95% CI: 3.87, 9.10) and increased their BMI of 2.47 kg/m^2 (95% CI: 1.45, 3.49; p=0.0001 for both). However, 50% (14/28) still had mild or more severe forms of malnutrition.

Conclusions: This cross-sectional survey of nutritional intake in patients undergoing TB treatment in a sanatorium demonstrates the difficulty in sufficiently meeting nutritional demands, even when providing nutritional support.

Keywords: tuberculosis; malnutrition; nutrition assessment; nutritional support; transients and migrants.

Word count: 2896 words

For peer review only

Article Summary

Strengths and limitations of the study

- Strong methodology tailored to the local diet to accurately assess nutritional intake.
- Limited in sample size and not a controlled study of a nutritional intervention.
- Costlier measures that more precisely estimate nutritional intake were not possible in this study.

For peer review only

Introduction

The interaction between malnutrition and tuberculosis (TB) has been long established [1]. Not only does malnutrition predispose individuals to TB infection, poor nutritional status and weight loss—particularly loss of lean body mass—are also associated with TB [2-4]. Malnutrition at the time of TB diagnosis increases mortality risk [2 5 6]. Inadequate nutritional intake is the most likely cause of poor nutritional status among patients with TB [1]. However, there is insufficient evidence to determine whether nutritional supplementation improves outcomes in patients with TB [3 7 8]. Even in situations where anti-tuberculous therapy and nutritional support is offered, many patients do not return to adequate nutritional status by the end of their treatment period [1 9]. However, in light of the strong link between malnutrition and TB, the WHO insists on nutritional assessment, counseling, and addressing nutritional deficits for patients with TB [3 5 8 10 11].

However, it is difficult to assess the nutritional intake of patients with TB to detect underlying nutritional deficits. Nutritional assessments commonly use 24-hour food recalls or food frequency questionnaires. These tools rely on food composition data to estimate nutrient intake. In low- and middle-income countries—where most of the global TB cases occur—the lack of nutrient composition data based on local diets leads to inaccurate estimates of nutrient intake [11]. Intake assessments that do not account for local diets and rely on patient recall may not accurately depict nutritional intake of TB patients in outpatient or community directly observed therapy (DOTS) programs [2 10].

Along the Myanmar-Thailand border, TB clinics provide services for migrants with TB via a unique, sanatorium model [12-14]. Nutritional support, in the way of rations, are included in the cadre of care and treatment services provided to patients who reside at the clinics for the duration of their treatment. Recent nutrition research has allowed a better characterization of the local diets along the border sufficient to determine nutritional content [15 16]. This cross-

For peer review only - http://bmjopen.bmj.com/site/about/guidelines.xhtml

sectional study seeks to quantify macro- and micronutrient intake of a sample of patients with TB along the border and to evaluate if meal rations adequately address patients' nutritional demands.

Methods

Setting: Tuberculosis Program along the border

Established in 2010, the Borderlands Health Foundation/Shoklo Malaria Research Unit (SMRU) Tuberculosis Program has provided a large breadth of services for TB patients through its sanatoria, including diagnostic and treatment procedures, housing, dietary, and psychosocial support services. By the end of January 2019, these clinics had provided treatment services to a total of 1925 patients, 1465 (76%) of whom had successfully completed the entirety of their treatment. At the time of this study, 118 patients were actively receiving treatment in TB clinics along the border. Patients are agricultural migrants from either side of the Myanmar-Thailand border and are predominantly of Burman or Karen ethnicity.

Study participants and design

To provide a representative sample of patients to enroll in this study, TB program patient records were reviewed to estimate the proportion of men and women, age, and those co-infected with HIV among the patient population. These proportions were then applied to the existing roster of patients currently under care. Thus, participants were selected through purposive sampling from participating TB clinics based on proportions representative of patient profiles from historical TB program records. Inclusion criteria included: TB confirmed by GeneXpert and/or AFB smear, with pulmonary or extrapulmonary infections, multi-drug resistant TB, or HIV co-infection. Participants were excluded if they were in treatment for less than 8 weeks, had a recent acute illness onset, unable to provide accurate 24-hour food recalls, or unable to provide consent/verbal assent. Participants meeting inclusion/exclusion criteria were approached and provided verbal assent for this study, as

For peer review only

informed consent had been waived for this audit of services in a vulnerable population where legal status needs to be kept confidential. Routine treatment was provided to all patients according to WHO guidelines. As thisThis was not a controlled study testing an intervention but assessing nutritional intake in a small subset of participants, so no sample size calculation was performed.

Nutrition support provided

Patients at TB clinics are provided daily meals for the duration of their treatment. Rations provided by the clinic include two full meals a day as well as refreshments (processed foods and liquids). Some participants were able to supplement their diet with food from outside sources in addition to the rations provided. In addition to daily meals, patients receive weekly rations which include biscuits, juice, and sweetened milk powders. Each meal consisted of a serving of rice, and a curry. Protein sources for meals come from animal sources everyday out of the week, with a predominance of chicken and fish. The meals were provided in timing and frequency according to local eating habits. While meal ingredients are budgeted for each individual patient, servings are not standardized. Chefs were locals from the communities and prepared meals according to local dietary customs and preferences.

Survey tools and validation

24-hour food recalls determined participant food intake and were adapted from a form validated for use in an observational cohort, "Molecular signature of Karen and Burmese pregnant women on the Thailand-Myanmar border," conducted at SMRU (ClinicalTrials.gov Identifier: NCT02797327) [15]. However, as portion size estimations—using visual and other size estimation aids or household utensil measures—are limited [17-19], caloric intake is often underestimated, such as for cereal grains among other food items [19]. To account for this potential limitation, dietitians advocate the use of multiple methods to estimate portion sizes in 24-hour food recalls such as hand measurements, which has been validated for accuracy

For peer review only - http://bmjopen.bmj.com/site/about/guidelines.xhtml

and appropriateness of this method for use in low resource settings [15]. In previous 24-hour food recalls in migrant clinics along the Myanmar-Thailand border, patients often underestimated their intake of rice—similar to studies in Africa [19]. Therefore, 24-hour food recalls have employed all methods at our disposal: standardized measuring instruments, hand measurements, and visual aids [17]. Using multiple methods of portion size estimation may enhance the accuracy of recall data.

Calculating Nutritional Intake

Following survey completion, food recall data was uploaded into a free, online nutrition data software (Cronometer, https://cronometer.com/). Nutrition data was calculated for the total intake, intake from rations alone, and intake excluding rice.

Recipes for all meals provided at the clinic as part of the rations were compiled by interviewing the cooks at the clinic and corroborating common recipes with several local staff at SMRU. This allowed compiling basic recipes for curries, soups, salads, stir fries, and pastes. Hence, any dish or meal reported as part of the rations allowed for the main ingredients to be determined and used in conjunction with the compiled recipes. These final recipes were then added to Cronometer.

As several ingredients in this community's diet were not available on Cronometer's database, nutrient data were obtained using the Mahidol University's Thai Food Composition Database and McGill University's Center for Indigenous Peoples' Nutrition and Environment Karen food composition database [20 21]. Nutritional information in these databases is often expressed in terms of nutrients per 100-gram sample and required recording densities of ingredients reported in recalls. This was determined either through direct measurement, matching densities to foods with similar descriptions, or averaging the densities of similar ingredients.

For peer review only

Anthropometric measurements and determining nutritional requirements

Anthropometric measurements were performed prior to beginning anti-tuberculous treatment and repeated for each participant immediately prior to conducting the 24-hour food recall and followed TB Program standard of procedures for weight and height assessment. Routinely calibrated mechanical scales were used to measure weight and height accurate to 500 grams and 5 mm, respectively. BMI was calculated and categorized into: severe malnutrition (< 16.0 kg/m^2), moderate malnutrition (16.0 – 16.9 kg/m^2), mild malnutrition (17.0 – 18.4 kg/m^2), normal (18.5 – 24.9 kg/m^2), and overweight (25.0 – 29.9 kg/m^2) [22]. Nutritional requirements were calculated for each individual patient and compared to participants' actual intake. For nutritional requirements of TB patients without comorbidities, a protein range of 1.2-1.5 g/kg Ideal Body Weight (IBW) was used. The energy range was 35-40 kcal/kg body weight. IBW was calculated using the Devine Equation (50kg +2.3kg X (Ht – 60 in) for men, and 45.5kg +2.3kg X (Ht - 60 in) for women), which was adapted for the metric system [23]. For patients with an HIV coinfection, the 35-40 kcal/kg range was increased by 20-30%. The estimated energy needs of the one participant with an above-the-knee amputation were reduced by 16%. National Academy of Medicine recommendations were used for carbohydrates and fats [24] and the Thai daily recommended intake (DRI) for micronutrients (vitamins A, B-complex, C, calcium, iron, sodium, zinc) [25].

Statistical Analysis

Analysis was conducted using Stata version 14 (StataCorp LP, College Station, TX, USA). Distributions of the data were assessed for normality, with descriptive data summarized using mean and 95% confidence intervals or median and inter-quartile range as appropriate. The primary outcome of this study was to evaluate participants' dietary intake and their individual nutritional requirements. The secondary outcome assessed weight gain and change in BMI from the start of anti-tuberculous treatment until the time of the survey.

For peer review only

For peer review only - http://bmjopen.bmj.com/site/about/guidelines.xhtml

Weight gain, change in BMI and differences in participant intake for all three macronutrients (protein, carbohydrates, and fats) and selected micronutrients were evaluated between gender and between type of ration using Student's t-test or Mann Whitney U test for normally distributed and non-parametrically data, respectively. Intake for each nutrient was then categorized as "dietary requirement achieved" or "not achieved" and compared between gender and type of ration with Fisher-exact test. The limited sample size prevented performing meaningful multivariable analysis.

Patient and public involvement in research

Prior to conducting this study, the research team involved the local population through a local community advisory board, the Tak Community Advisory Board, Thailand. Comprised of community leaders, they advised on study design and implementation and approved the study (TCAB 201903). No study participants were directly involved in the design of the study. Although we have not disseminated findings to the study participants who had completed treatment and returned to their communities by the end of the study, study findings have been disseminated to staff to inform meal rations provided by the TB clinics and to inform nutrition counseling for future TB patients receiving care at the clinic.

Results

Table 1 summarizes participants' basic demographics, treatment, and anthropometric variables for all 28 participants. More men were included in the analysis (20/28, 71.4% vs 8/28, 28.6%). Additionally, the men were significantly older than women (52.4 vs 37.6 years, p=0.021). A total of 17.9% (5/28) of participants were coinfected with HIV. None of the participants reported substance use such as alcohol, cigarettes, or other drugs. Just over half the participants (57.1%, 16/28) ate only the rations provided by the clinic. No participants had MDRTB, and only one male participant (1/20, 5%) had an extrapulmonary form of TB. At diagnosis, men were significantly taller (160.7 vs 150.8 cm, p=0.002) and heavier (43.2 vs

For peer review only - http://bmjopen.bmj.com/site/about/guidelines.xhtml

36.9 kg, p=0.011) than women, but there were no significant differences for BMI (16.7 vs 16.3 kg/m^2, p=0.630). All participants were currently receiving treatment with a mean treatment length at the time of survey of 20.7 weeks (95% CI: 16.5-24.8). Pairwise comparisons show 22/28 (78.6%) participants had increased weight and BMI from diagnosis to the time of the survey; with an average gain of 6.48 kg (95% CI: 3.87, 9.10) and 2.47 kg/m^2 (95% CI: 1.45, 3.49) per participant (p=0.0001 for both). By the time of the survey, the weight of men and women were no longer significantly different (51.0 vs 44.0 kg, p=0.115). This improvement in weight and BMI is reflected in the shift of malnutrition category from diagnosis to the time of survey: at the time of diagnosis 89% (25/28) of participants had mild or more severe forms of malnutrition compared to 50% (14/28) of participants at the time of the survey (p=0.22).

Table 2 outlines the average nutritional intake stratified by sex and whether participants ate rations only. There were no significant differences in total macro- or micronutrient intake when comparing men to women, or patients eating only the rations provided compared to those supplementing the rations received.

Table 3 highlights the proportion of participants meeting their recommended daily allowances. Participants' diets were low in protein, fats, and nearly all vitamins, but were high in salt content. A significantly higher proportion of women met their overall caloric and vitamin C requirements (p=0.010, p=0.044 respectively), while a higher proportion of men met their iron intake requirements (p=0.011). Meeting the remaining dietary requirements did not differ significantly between the sexes or having a ration-only diet.

Discussion

This study presents findings from a cross-sectional survey of TB patients undergoing treatment while residing at a sanatorium-style clinic along the Myanmar-Thailand border. The most notable finding in this study is the difficulty patients had reaching their nutrition

For peer review only - http://bmjopen.bmj.com/site/about/guidelines.xhtml

requirements. TB patients supplementing the rations provided by the clinic were no more likely to meet their requirements than those eating only the provided rations. Frequently, total calories and protein requirements were not met, and although carbohydrates approached the needed requirements, most of the total carbohydrates were acquired from rice, reflecting a lack of dietary diversity. This lack of dietary diversity is likely responsible for participants' inability to meet most micronutrient requirements. Similar to other study findings, although there was some weight gain, improvement in BMI, and malnutrition status, this fell below the level desired [1 8 9]. Overall intake of almost all important micronutrients was limited, with sodium intake often being too high—even for patients eating only the rations provided., further suggesting that participant diets lack the diversity required to meet nutritional recommendations.

These inadequacies in nutrient intake among TB patients echoes findings in other studies [1 2 7 9 26]. This study adds to the literature by including robust data on the nutritional content of local diets; provides strong nutritional intake data given the sanatorium-style setting; and provides baseline data for a vulnerable, migrant population that likely has worse nutritional status upon diagnosis than may be seen in other populations. Compared to other settings where nutrient intake was estimated in accordance to the local diet, TB patients in Georgia were able to manage high caloric intake for follow-up in an outpatient setting [10].

In this setting in Asia, it may be useful to highlight some recommendations for increasing nutritional intake: a regular source of protein; small yet regular meals; and locally sourced fresh fruits and vegetables or other snacks low in sodium and saturated fats that provide additional micronutrient supplementation. These recommendations should be considered alongside regular dietary counseling and support—if human resources can be allocated—as recommended by the WHO [8]. Not only are patients recommended to have an assessment when enrolled for care but support may also be important at the beginning stages of drug

For peer review only - http://bmjopen.bmj.com/site/about/guidelines.xhtml

treatment, when side effects such as nausea, vomiting, and loss of appetite can interfere with early nutritional gains [26]. A culturally-tailored counseling program, representative of the local diet, can help inform TB patients about the importance of a diverse diet, especially when undergoing treatment and as local community awareness about good nutrition may be limited. Including family members in these discussions would be helpful as family members often stay with patients in the clinic. Such counseling can also help inform TB patients (and their caregivers) of good nutritional foods should they be able to purchase foods to supplement their rations.

This study is not without limitations. As this was not a controlled intervention, we did not perform a sample size calculation and instead elected to take a small subset of TB patients to participate in this study such that it could be conducted feasibly and quickly with the staff on hand. In addition to its small size, this study is limited as it is cross-sectional in design—although this is the design reported by most studies of this nature. With further funding, future studies will assess nutritional intake in addition to blood and serum measures of nutritional status among a larger sample of patients, as the free software we used for this study limited our analysis to only 6 micronutrients. The comparisons drawn in this sample are limited as well, given the broad inclusion criteria (e.g., including HIV co-infected) that were chosen to include a more representative sample of patients from this border region to inform future nutritional interventions. A further limitation in participants assessed in this study is that relapsed TB patients and those with comorbidities such as diabetes mellitus were not included, as their treatment and nutritional recommendations may differ from what is commonly prescribed for TB patients. The significant difference in gender in our study, however, is representative of the greater number of TB infections among men in the general population. Future studies may also benefit from using additional anthropometric measures, especially those more sensitive in detecting undernutrition [6 9 27 28] and account for disease

For peer review only

For peer review only - http://bmjopen.bmj.com/site/about/guidelines.xhtml

severity [2,5]. Some limitation is inherent to the methods used such as not performing serial food recall surveys, measurement intake accuracy, and recall bias[17,18,29], and aligning food descriptions from the local diet with food composition databases. However, the tools used have been developed across different nutritional studies in this area and have allowed for validating methods across different segments of the local migrant populations.

Conclusions

This cross-sectional survey of TB patients residing in a sanatorium along the Myanmar-Thailand border demonstrates the difficulty in sufficiently meeting nutritional demands, even when providing nutritional support.

Abbreviations

BMI	Body-mass index
DRI	Daily recommended intake
SMRU	Shoklo Malaria Research Unit
TB	Tuberculosis
WHO	World Health Organization

Acknowledgements

We are grateful for the patients who volunteered to be a part of this study. We would like to acknowledge the staff working in the TB department at SMRU, for their help and support with logistics, materials, and other needs. We would like to thank the local SMRU staff that helped with recipes and understanding local diets, including the cooks at the TB sanatorium. We would like to thank Wolfgang Stuetz for feedback on drafts of this manuscript.

Ethics approval

This study received ethical approval from the Research Ethics Committee of the Faculty of Medicine, Chiang Mai University (FAM-2562-06197) in accordance with the Declaration of Helsinki. Informed verbal assent was given by all participants prior to survey, with written informed consent waived by the Research Ethics Committee of Chiang Mai University.

Consent for publication

Not applicable.

For peer review only

Data availability statement

The datasets generated and/or analyzed during the current study are not publicly available due to privacy/safety for migrant participants, but are available from the corresponding author on reasonable request.

Competing interests

The authors declare that they have no competing interests.

Funding

This research was funded in whole, or in part, by the Wellcome Trust Thailand/Laos Major Overseas Program Renewal [grant number 106698]. For the purpose of open access, the author has applied a CC BY public copyright licence to any Author Accepted Manuscript version arising from this submission. The funding body had no role in the design of the study and collection, analysis, and interpretation of data and in writing the manuscript.

Author contributions

KD, AHH, MVD, CA, VIC, FN: study conception, design; KD, AHH, LLK, WPPH, HKKA, TB: data collection and study implementation; KD, AHH, VIC: data analysis/interpretation; All authors critically revised the manuscript and approved the final version submitted and agree to be accountable for this work.

References

1. Macallan DC. Malnutrition in tuberculosis. *Diagnostic microbiology and infectious disease* 1999;34(2):153-7.
2. Mupere E, Parraga IM, Tisch DJ, et al. Low nutrient intake among adult women and patients with severe tuberculosis disease in Uganda: a cross-sectional study. *BMC Public Health* 2012;12:1050.
3. Sinclair D, Abba K, Grobler L, et al. Nutritional supplements for people being treated for active tuberculosis. *Cochrane Database of Systematic Reviews* 2011;11:CD006086.
4. White LV, Edwards T, Lee N, et al. Patterns and predictors of co-morbidities in Tuberculosis: A cross-sectional study in the Philippines. *Scientific Reports* 2020;10:4100.
5. Miyata S, Tanaka M, Ihaku D. The prognostic significance of nutritional status using malnutrition universal screening tool in patients with pulmonary tuberculosis. *Nutr J* 2013;12:42.

For peer review only

6. Zachariah R, Spielmann MP, Harries AD, et al. Moderate to severe malnutrition in patients with tuberculosis is a risk factor associated with early death. *Transactions of the Royal Society of Tropical Medicine and Hygiene* 2002;96(3):291-4.
7. Rudolph M, Kroll F, Beery M, et al. A pilot study assessing the impact of a fortified supplementary food on the health and well-being of creche children and adult TB patients in South Africa. *PLoS ONE [Electronic Resource]* 2013;8(1):e55544.
8. World Health Organization. Nutritional care and support for patients with tuberculosis. Geneva: WHO, 2013.
9. Bacelo AC, Ramalho A, Brasil PE, et al. Nutritional supplementation is a necessary complement to dietary counseling among tuberculosis and tuberculosis-HIV patients. *PloS one* 2015;10(8):e0134785.
10. Frediani JK, Sanikidze E, Kipiani M, et al. Macronutrient intake and body composition changes during anti-tuberculosis therapy in adults. *Clin Nutr* 2016;35(1):205-12.
11. Frediani JK, Tukvadze N, Sanikidze E, et al. A culture-specific nutrient intake assessment instrument in patients with pulmonary tuberculosis. *Clin Nutr* 2013;32(6):1023-8.
12. Thi SS, Parker DM, Swe LL, et al. Migration histories of multidrug-resistant tuberculosis patients from the Thailand-Myanmar border, 2012-2014. *Int J Tuberc Lung Dis* 2017;21(7):753-58.
13. Tschirhart N, Nosten F, Foster AM. Migrant tuberculosis patient needs and health system response along the Thailand-Myanmar border. *Health Policy Planning* 2017;32:1212-19.
14. Tschirhart N, Sein T, Nosten F, et al. Migrant and refugee patient perspectives on travel and tuberculosis along the Thailand-Myanmar border: a qualitative study. *PLOS ONE* 2016;11(8):e0160222.
15. Brummaier T, Kabeer BSA, Lindow S, et al. A prospective cohort for the investigation of alteration in temporal transcriptional and microbiome trajectories preceding preterm birth: a study protocol. *BMJ Open* 2018;9:e023417.
16. Brummaier T, Kabeer BSA, Wilaisrisak P, et al. Cohort profile: molecular signature in pregnancy (MSP): longitudinal high-frequency sampling to characterise cross-omic trajectories in pregnancy in a resource-constrained setting. *BMJ Open* 2020;10:e041631.
17. Amoutzopoulos B, Paige P, Roberts C, et al. Portion size estimation in dietary assessment: a systematic review of existing tools, their strengths and limitations. *Nutrition Reviews* 2020;78(11)
18. Gibson AA, Hsu MS, Rangan AM, et al. Accuracy of hands *v.* household measures as portion size estimation aids. *J Nutritional Sci* 2016;5(e29)
19. Ogunbode AM, Owolabi MO, Ogunbode OO, et al. What's in your hands? A systematic review of dietary assessment methods and estimation of food sizes in a primary care clinic. *J Med Tropics* 2018;20(2):93.
20. Mahidol University, Institute of Nutrition. Thai Food Composition Database 2015 [Available from: http://www.inmu.mahidol.ac.th/thaifcd.
21. McGill University, Environment CfIPNa. CINE Global Health Case Study - Karen 2019 [Available from: https://www.mcgill.ca/cine/resources/data/karen.
22. World Health Organization. Management of severe malnutrition: a manual for physicians and other senior health workers. Geneva: WHO, 1999.
23. Peterson CM, Thomas DM, Blackburn GL, et al. Universal equation for estimating ideal body weight and body weight at any BMI. *Am J Clin Nutr* 2016;103(5):1197-203.
24. National Academy of Medicine. Dietary reference intakes for energy, carbohydrate, fiber, fat, fatty acids, cholesterol, protein, and amino acids. Washington, D.C.: National Academy of Medicine, 2005.

For peer review only

25. Bureau of Nutrition, Department of Health, Thailand Ministry of Public Health. Dietary reference intake for Thais 2020. Bangkok: Ministry of Public Health, 2020.
26. Bacelo AC, do Brasil PE, Cople-Rodrigues CD, et al. Dietary counseling adherence during tuberculosis treatment: A longitudinal study. *Clin Nutr ESPEN* 2017;17:44-53.
27. Lee N, White LV, Marin FP, et al. Mid-upper arm circumference predicts death in adult patients admitted to a TB ward in the Philippines: a prospective cohort study. *PLOS ONE* 2019;14(6):e0218193.
28. White LV, Lee N, Marin FP, et al. Performance of alternative measures to body mass index in the assessment of moderate and severe under-nutrition among acutely unwell patients hospitalized in a TB ward in the Philippines: a cross-sectional study. *PLOS ONE* 2019;14(5):e0215968.
29. Péter S, Saris WHM, Mathers J, et al. Nutrient status assessment in individuals and populations for healthy aging—statement from an expert workshop. *Nutrients* 2015;7(12):10491-500.

For peer review only

Table 1. Basic demographic, treatment, and anthropometric variables.

	All participants (N=28)	Male[1] (20, 71.4%)	Female (8 (28.6%)	
Variables				
Age (mean, 95% CI, years)	48.2 (42.1, 54.2)	52.4 (45.9, 58.9)	37.6 (24.8, 50.5)	p=0.021
Ration only (n, %)				p=0.69
Yes	16 (57.1)	12 (60.0)	4 (50.0)	
No	12 (42.9)	8 (40.0)	4 (50.0)	
HIV coinfection (n, %)				p=1.00
Positive	5 (17.9)	4 (20.0)	1 (12.5)	
Negative	23 (82.1)	16 (80.0)	7 (87.2)	
Treatment length at time of survey (mean, 95% CI, weeks)	20.7 (16.5, 24.8)	20.1 (15.4, 24.8)	22.2 (11.4, 32.9)	p=0.628
Height (mean, 95% CI, cm)	157.8 (154.6, 161.1)	160.7 (157.4, 163.9)	150.8 (144.4, 157.2)	p=0.003
Weight at diagnosis (mean, 95% CI, kg)	41.4 (39.1, 43.8)	43.2 (40.7, 45.8)	36.9 (31.9, 41.8)	p=0.011
Weight at survey (mean, 95% CI, kg)	49.0 (44.9, 53.0)	51.0 (46.3, 55.6)	44.0 (35.1, 52.9)	p=0.115
BMI at diagnosis (mean, 95% CI, kg/m^2)	16.1 (15.7, 17.5)	16.7 (15.7, 17.8)	16.3 (13.8, 18.7)	p=0.630
BMI at survey (mean, 95% CI, kg/m^2)	19.5 (18.3, 20.7)	19.7 (18.3, 21.1)	19.1 (16.3, 21.9)	p=0.682

[1]Includes one participant with an amputation of one leg and one with extrapulmonary TB.

Table 2. Nutrition intake reported as median (IQR).

	Totals (N=28)	Male (n=20)	Female (n=8)	Ration only (n=16)	Ration+ (n=12)
Calories (kcal)	2153.5 (1764.0, 3118.0)	2065.0 (1487.5, 3118.0)	2325.0 (1986.5, 3158.5)	2035.0 (1454.5, 2721.5)	2153.5 (1968.0, 3638.0)
Calories from rice (kcal)	967.0 (789.0, 1396.5)	967.0 (842.0, 1450.5)	967.0 (698.5, 1396.5)	967.0 (644.5, 1397.0)	967.5 (789.0, 1396.5)
Protein (kcal)	277.6 (192.6, 371.6)	268.6 (178.4, 371.6)	305.8 (225.8, 421.4)	268.6 (163.6, 360.8)	302.4 (225.8, 422.0)
Carbohydrates (kcal)	1240.4 (1061.6, 1718.0)	1248.0 (1025.6, 1891.0)	1231.8 (1098.0, 1522.0)	1160.2 (1008.6, 1718.0)	1313.8 (1173.8, 1791.8)
Fats (kcal)	540.9 (385.7, 797.4)	455.9 (383.9, 736.7)	756.9 (526.5, 982.4)	464.9 (356.4, 711.0)	736.7 (403.2, 947.7)

For peer review only

Vitamin A (mcg RAE)	293.8 (150.5, 459.4)	226.8 (150.5, 459.4)	360.7 (150.6, 494.2)	226.8 (1510.5, 425.6)	398.8 (141.9, 502.6)
Vitamin B1 (mg)	1.0 (0.7, 1.5)	1.0 (0.8, 1.5)	1.1 (0.7, 1.5)	1.1 (0.8, 1.3)	0.9 (0.7, 1.7)
Vitamin B2 (mg)	1.2 (0.7, 1.6)	1.2 (0.7, 1.6)	1.2 (0.6, 1.7)	1.1 (0.7, 1.6)	1.3 (0.8, 2.1)
Vitamin B3 (mg)	24.2 (17.6, 36.1)	25.7 (16.9, 38.2)	23.8 (20.0, 32.5)	25.9 (16.9, 38.2)	24.0 (19.4, 32.8)
Vitamin B5 (mg)	3.5 (1.3, 4.4)	3.0 (1.3, 4.2)	3.7 (2.1, 5.6)	3.4 (1.6, 4.2)	3.7 (0.9, 4.9)
Vitamin B6 (mg)	1.7 (1.0, 2.3)	1.65 (1.0, 2.3)	1.7 (0.9, 2.1)	1.9 (1.0, 2.2)	1.6 (1.0, 2.5)
Vitamin B9 (mcg)	234.0 (116.5, 303.4)	205.1 (116.5, 291.9)	268.9 (142.1, 324.0	203.2 (116.5, 293.7)	280.3 (119.4, 321.5)
Vitamin B12 (mcg)	4.3 (1.0, 6.0)	4.1 (1.0, 5.3)	5.8 (1.7, 8.0)	3.5 (1.5, 5.9)	4.7 (0.8, 6.4)
Vitamin C (mg)	65.2 (48.4, 132.7)	59.8 (43.1, 111.0)	112.7 (73.8, 170.8)	62.2 (47.7, 126.9)	79.5 (48.4, 132.7)
Calcium (mg)	586.5 (365.8, 906.4)	619.9 (412.2, 906.4)	465.5 (292.5, 951.8)	496.0 (332.5, 690.1)	642.4 (417.2, 1138.8)
Iron (mg)	14.0 (8.0, 18.4)	14.7 (8.6, 18.4)	10.9 (6.5, 18.2)	11.0 (7.2, 19.4)	15.5 (10.7, 18.4)
Sodium (mg)	4011.9 (2481.2, 5564.5)	4011.9 (2283.2, 5359.7)	4419.4 (3032.8, 5759.0)	3727.5 (2848.5, 5359.7)	4429.6 (2230.9, 6245.9)
Zinc (mg)	6.4 (3.1, 10.0)	5.8 (3.1, 10.0)	8.4 (3.3, 12.0)	5.8 (4.2, 9.0)	8.1 (1.4, 10.7)

Thai Dietary Reference Intake: vitamin A: 700 mcg RAE males ≥ 19 y, 600 mcg RAE females ≥ 19 y; B1: 1.2 mg males ≥ 13 y, 1.1 mg females ≥ 19 y; B2: 1.3 mg males ≥13 y, 1.1 mg females ≥ 19 y; B3: 16 mg males ≥ 13 y, 14 mg females ≥ 13 y; B5: 5 mg males, females ≥ 13 y; B6: 1.3 mg males 19-50 y, 1.7 mg males ≥ 51 y, 1.3 mg females 19-50 y, 1.5 mg females ≥ 51 y; B12: 2.4 mcg males, females ≥ 16 y; vitamin C: 100 mg males ≥ 19 y, 85 mg females ≥ 19 y; calcium 800 mcg males/females 19-50 y, 1000 mcg males/females ≥ 51y; folate: 300 mcg males/females ≥ 13 y; iron 11.5 mg males 19 60 y, 11 mg males ≥ 61 y, 20 mg females 19-50 y, 10 mg females ≥ 51 y; sodium: all participants require less than 2500 mg per day; zinc: 11.6 mg males 19-30 y, 10.9 mg 31-70 y, 10.3 mg males ≥ 71 y, 9.7 mg females 19-30 y, 9.2 mg females 31-60; 8.6 mg females ≥ 61 y [24].

Table 3. Percentage (%) of participants meeting their daily requirements.

	Total (N=28)	Male (n=20)	Female (n=8)	Ration only (n=16)	Ration+ (n=12)
Calories[1]Calories[1]	60.7	45.0	100.0[2]	56.3	66.7
Protein	53.6	45.0	75.0	43.8	66.7

For peer review only - http://bmjopen.bmj.com/site/about/guidelines.xhtml

Carbohydrates	89.3	95.0	75.0	93.8	83.3
Fats	60.7	60.0	62.5	56.3	66.7
Vitamin A	3.6	0.0	12.5	6.3	0.0
Vitamin B1	42.9	40.0	50.0	43.8	41.7
Vitamin B2	53.6	55.0	50.0	50.0	58.3
Vitamin B3	89.3	85.0	100.0	81.3	100.0
Vitamin B5	17.9	10.0	37.5	12.5	25.0
Vitamin B6	57.1	55.0	62.5	56.3	58.3
Vitamin B9	25.0	20.0	37.5	25.0	25.0
Vitamin B12	64.3	60.0	75.0	62.5	66.7
Vitamin C[2]C[2]	42.9	30.0	75.0[3]	37.5	50.0
Calcium	21.4	20.0	25.0	12.5	33.3
Iron[3]Iron[3]	64.3	80.0[4]	25.0	62.5	66.7
Sodium[4]Sodium[4]	25.0	30.0	12.5	18.8	33.3
Zinc	21.4	20.0	25.0	18.8	25.0

[1]Higher proportion of females met caloric requirements than males (p=0.010, Fisher's exact test).
[2]Higher proportion of females met vitamin C requirements than males (p=0.044, Fisher's exact test).
[3]Higher proportion of males met iron intake requirements than females (p=0.011, Fisher's exact test).
[4]Proportion[4]Proportion of participants with sodium consumption below 2500 mg/24 h vs those with high sodium intake.

For peer review only

Reporting checklist for cross sectional study.

Based on the STROBE cross sectional guidelines.

Instructions to authors

Complete this checklist by entering the page numbers from your manuscript where readers will find each of the items listed below.

Your article may not currently address all the items on the checklist. Please modify your text to include the missing information. If you are certain that an item does not apply, please write "n/a" and provide a short explanation.

Upload your completed checklist as an extra file when you submit to a journal.

In your methods section, say that you used the STROBE cross sectionalreporting guidelines, and cite them as:

von Elm E, Altman DG, Egger M, Pocock SJ, Gotzsche PC, Vandenbroucke JP. The Strengthening the Reporting of Observational Studies in Epidemiology (STROBE) Statement: guidelines for reporting observational studies.

For peer review only

		Reporting Item	Page Number
**Title and abstract**			
Title	#1a	Indicate the study's design with a commonly used term in the title or the abstract	1
Abstract	#1b	Provide in the abstract an informative and balanced summary of what was done and what was found	2
**Introduction**			
Background / rationale	#2	Explain the scientific background and rationale for the investigation being reported	4
Objectives	#3	State specific objectives, including any prespecified hypotheses	4
**Methods**			
Study design	#4	Present key elements of study design early in the paper	5
Setting	#5	Describe the setting, locations, and relevant dates, including periods of	5

		recruitment, exposure, follow-up, and data collection	
Eligibility criteria	#6a	Give the eligibility criteria, and the sources and methods of selection of participants.	5
	#7	Clearly define all outcomes, exposures, predictors, potential confounders, and effect modifiers. Give diagnostic criteria, if applicable	6
Data sources / measurement	#8	For each variable of interest give sources of data and details of methods of assessment (measurement). Describe comparability of assessment methods if there is more than one group. Give information separately for for exposed and unexposed groups if applicable.	7
Bias	#9	Describe any efforts to address potential sources of bias	7
Study size	#10	Explain how the study size was arrived at	5
Quantitative variables	#11	Explain how quantitative variables were handled in the analyses. If applicable, describe which groupings were chosen, and why	8
Statistical methods	#12a	Describe all statistical methods, including those used to control for confounding	8
Statistical methods	#12b	Describe any methods used to examine subgroups and interactions	8
Statistical methods	#12c	Explain how missing data were addressed	NA
Statistical methods	#12d	If applicable, describe analytical methods taking account of sampling strategy	NA
Statistical methods	#12e	Describe any sensitivity analyses	NA
**Results**			
Participants	#13a	Report numbers of individuals at each stage of study—eg numbers potentially eligible, examined for eligibility, confirmed eligible, included in the study, completing follow-up, and analysed. Give information separately for for exposed and unexposed groups if applicable.	8
Participants	#13b	Give reasons for non-participation at each stage	NA
Participants	#13c	Consider use of a flow diagram	NA

For peer review only

Descriptive data	#14a	Give characteristics of study participants (eg demographic, clinical, social) and information on exposures and potential confounders. Give information separately for exposed and unexposed groups if applicable.	8
Descriptive data	#14b	Indicate number of participants with missing data for each variable of interest	NA
Outcome data	#15	Report numbers of outcome events or summary measures. Give information separately for exposed and unexposed groups if applicable.	9
Main results	#16a	Give unadjusted estimates and, if applicable, confounder-adjusted estimates and their precision (eg, 95% confidence interval). Make clear which confounders were adjusted for and why they were included	9
Main results	#16b	Report category boundaries when continuous variables were categorized	9
Main results	#16c	If relevant, consider translating estimates of relative risk into absolute risk for a meaningful time period	9
Other analyses	#17	Report other analyses done—e.g., analyses of subgroups and interactions, and sensitivity analyses	NA
**Discussion**			
Key results	#18	Summarise key results with reference to study objectives	9
Limitations	#19	Discuss limitations of the study, taking into account sources of potential bias or imprecision. Discuss both direction and magnitude of any potential bias.	11
Interpretation	#20	Give a cautious overall interpretation considering objectives, limitations, multiplicity of analyses, results from similar studies, and other relevant evidence.	11
Generalisability	#21	Discuss the generalisability (external validity) of the study results	11
**Other Information**			
Funding	#22	Give the source of funding and the role of the funders for the present study and, if applicable, for the original study on which the present article is based	12

The STROBE checklist is distributed under the terms of the Creative Commons Attribution License CC-BY.
This checklist was completed on 30. April 2021 using https://www.goodreports.org/, a tool made by the EQUATOR Network in collaboration with Penelope.ai

For peer review only
